# ADQ-YOLOv8m: a precise detection model of sugarcane disease in complex environment

**DOI:** 10.3389/fpls.2025.1669825

**Published:** 2025-10-02

**Authors:** Zhaowen Li, Jihong Sun, Ying Yang, Jiangquan Chen, Qian Yu, Zheng Zhou, Yan Yang, Tao Yin, Haokai Zhang, Ye Qian

**Affiliations:** ^1^ College of Big Data, Yunnan Agricultural University, Kunming, China; ^2^ Key Laboratory of Artifcial Intelligence in Yunnan Province, Kunming University of Science and Technology, Kunming, Yunnan, China; ^3^ School of Information Engineering, Kunming University, Kunming, China; ^4^ College of Animal Veterinary Medicine, Yunnan Agricultural University, Kunming, China; ^5^ National Pilot School of Software, Yunnan University, Kunming, China; ^6^ Scientific and Technological Achievements Transfer and Transformation Center, Yunnan Provincial Academy Of Science And Technology, Kunming, China; ^7^ Engineering College, China Agricultural University, Beijing, China

**Keywords:** complex environment, sugarcane diseases, YOLOv8, precise detection, generalization ability

## Abstract

**Introduction:**

Current research on sugarcane disease identification primarily focuses on a limited number of typical diseases, often constrained by specific target groups or conditions. To address this, we propose an enhanced ADQ-YOLOv8m model based on the YOLOv8m framework, enabling precise detection of sugarcane diseases.

**Methods:**

The detection head is modified to a Dynamic Head to enhance feature representation capabilities. Following the Detect module, we introduce the ATSS dynamic label assignment strategy and the QFocalLoss loss function to address issues such as class imbalance, thereby bolstering the model’s feature representation capabilities.

**Results:**

Experimental results demonstrate that ADQ-YOLOv8m outperforms nine other mainstream object detection models, achieving precision, recall, mAP50, mAP50-95, and F1 scores of 86.90%, 85.40%, 90.00%, 77.40%, and 86.00%, respectively.

**Discussion:**

Finally, comprehensive evaluation of the ADQ-YOLOv8m model’s performance is conducted using visual analysis of image predictions and cross-scenario adaptability testing. The experimental results indicate that the proposed model excels in multi-objective processing and demonstrates strong generalization capabilities, suitable for scenarios involving multiple objectives, multiple categories, and class imbalance. The detection method proposed exhibits excellent detection performance and potential, providing robust support for the development of intelligent sugarcane cultivation and disease control.

## 1 Introduction

Sugarcane, as an important economic crop, plays a pivotal role in agricultural production. During its growth cycle, it demands a large amount of fertilizer, along with sufficient arable land resources, irrigation water sources, and abundant nutrient supply. In China, the production of sugarcane directly affects the total sugar output, with over 90% of sugar coming from this crop (Liu et al., 2024).

However, in the process of sugarcane cultivation, disease issues have become a significant factor constraining its industrial development. Existing research has shown that plant diseases are one of the key factors threatening crop productivity and quality. Globally, 20%-40% of agricultural productivity losses are caused by plant diseases (Qaadan et al., 2025). Specifically in sugarcane cultivation, the occurrence of diseases can have severe negative impacts on the sugarcane industry (Rott et al., 2015). Infected sugarcane plants often exhibit symptoms such as slow growth, wilted leaves, softened stems, and decreased yields. These symptoms not only significantly reduce the yield and quality of sugarcane but may even lead to plant death, causing huge economic losses to growers (Huang et al., 2018). Therefore, accurately identifying sugarcane diseases is of utmost importance. Efficient and precise disease identification can provide farmers with scientific evidence, enabling them to take effective prevention and control measures, thereby reducing economic losses, curbing the further spread of diseases, and ensuring the sustainable development of the sugarcane industry.

Deep learning and image processing technologies (LeCun et al., 2015; Demilie, 2024; Ritharson et al., 2024; Li et al., 2024) have become indispensable tools in the agricultural sector, particularly in the identification and classification of crop leaf diseases (Hang et al., 2019). Researchers have been exploring various methods to enhance plant disease recognition. Yuzhi Wang et al. proposed a new model combining a masked autoencoder (MAE) and a convolutional block attention module (CBAM). Through experiments on 21 leaf diseases of five crops, namely potatoes, corn, tomatoes, cashews, and cassava, this model achieved accurate and rapid detection of plant dis-ease categories (Wang et al., 2024). Jianping Yao et al. introduced a new model called Generalized Stacked Multi-Output CNN (GSMo-CNN) and proposed the hypothesis that using a single model for two tasks can be comparable to or better than using two models for each task (Yao et al., 2024). C. Ashwini et al. proposed a hybrid 3DCNN-RNN model optimized by the Joint Search Whale Optimization Algorithm (JSWOA). Simulation results showed that the proposed hybrid model achieved a performance of over 90% in predicting various corn leaf categories on two datasets (Ashwini and Sellam, 2024). Imane Bouacida et al. addressed the issue of deep learning models lacking robustness and generalization ability when faced with new crops and disease types not included in the training dataset. They proposed a novel system based on deep learning, emphasizing the core goal of focusing on identifying the disease itself rather than solely relying on the appearance of diseased leaves (Bouacida et al., 2025). Lobna M. Abouelmagd et al. utilized an optimized Capsule Neural Network (CapsNet) to detect and classify ten tomato leaf diseases using standard dataset images. The research results highlighted the potential of CapsNet as an alternative to CNNs (Abouelmagd et al., 2024). Marriam Nawaz et al. introduced a novel and effective deep learning model called CoffeeNet to overcome the challenges of various image distortions and the similarity between healthy and diseased parts of inspection samples in coffee disease recognition (Nawaz et al., 2024). Anuja Bhargava et al. emphasized the importance of computer vision and artificial intelligence for automatic monitoring of plant leaf health and disease detection, highlighting the role of molecular diagnostic tools and segmentation algorithms in improving agricultural processes (A et al., 2024).

Crop disease target detection has always been an important field in agricultural research, with various studies focusing on utilizing advanced technologies to improve detection accuracy and efficiency. Xuewei Wang et al. proposed YOLOv8n-vegetable, which made multiple improvements and optimizations to the YOLOv8n model to enhance its effectiveness, better preserve the fused feature information, and thus enhance vegetable disease detection in greenhouse environments. This improvement resulted in a 6.46% increase in mean average precision (mAP) compared to the original model when applied to a self-built vegetable disease detection dataset under green-house conditions (Wang and Liu, 2024). Dong Cong Trinh et al. proposed an improved YOLOv8 model based on EIoU loss and α-IoU loss to enhance the performance of the rice leaf disease detection system (Trinh et al., 2024). Sasikala Vallabhajosyula et al. introduced a novel hierarchical residual visual transformer, utilizing an improved Vision Transformer and ResNet9 model to aid in the early detection of leaf diseases (Vallabhajosyula et al., 2024). Meng Lv et al. innovatively incorporated attention mechanisms and modules containing transformer encoders into YOLOV5, resulting in YOLOV5-CBAM-C3TR for apple leaf disease detection. When applied to the detection of two very similar diseases (including Alternaria leaf spot and gray leaf spot), it achieved accuracies of 93.1% and 89.6%, respectively (Lv and Su, 2023). Mazin Abed Mohammed et al. addressed the challenge of collecting plant disease data from land distributed across different regions by utilizing a deep neural network with transfer learning to propose plant disease detection based on edge cloud remote sens-ing data (M et al., 2024). Abudukelimu Abulizi et al. introduced an improved tomato leaf disease detection method, DM-YOLO, based on the YOLOv9 algorithm. When evaluated on the tomato leaf disease dataset, the model achieved a precision (P) of 92.5%, with average precision (AP) and mean average precision (mAP) of 95.1% and 86.4%, respectively (Abulizi et al., 2024). Jianlong Wang et al. proposed a lightweight method, LCGSC-YOLO, to address issues such as the large number of learning parameters and complex scenarios in apple leaf disease detection, aiming to improve the detection accuracy decline caused by model lightweighting (Wang et al., 2024). Arun Kumar Sangaiah et al. proposed a deep learning architecture, UAV T-YOLO-RICE, suitable for application in aerial computing dronemounted intelligence, achieving a test average precision (mAP) of 86% (A et al., 2024).

In the field of sugarcane disease identification and detection, researchers have explored deep learning methods, particularly convolutional neural networks (CNNs), for accurate and timely detection of sugarcane diseases (Srinivasan et al., 2025; Bala and Bansal, 2024; Bao et al., 2024). These methods involve utilizing high-resolution images of affected sugarcane parts, which undergo meticu-lous preprocessing to enhance key features and minimize noise interference. Furthermore, indepth research has been conducted on developing and evaluating deep learning-based methods using the EfficientNet model to robustly detect diseases in sugarcane leaves (Kunduracıoğlu and Paçal, 2024). Sakshi Srivastava et al. proposed a novel deep learning frame-work method that detects the presence of diseases in sugarcane plants by analyzing their leaves, stems, and colors. Using VGG-16 as the feature extractor and SVM as the classifier, they achieved an AUC of 90.2% (Srivastava et al., 2020). Dong Bao et al. utilized hyperspectral imaging and a spectral-spatial attention deep neural network to detect early signs of smut and mosaic diseases in sugarcane. Experimental results showed that the deep neural network model effectively extracted hyperspectral images containing features useful for early detection of the two target sugarcane diseases. The detection accuracy for both diseases was over 90% before visible symptoms appeared (Bao et al., 2024). Abirami Kuppusamy et al. integrated the Vision Transformer architecture with Hybrid Shifted Windows to propose a novel automatic classification method for sugarcane leaf diseases, achieving a disease detection accuracy of up to 98.5% (Kuppusamy et al., 2024a). Jihong Sun et al. optimized the YOLOv8 model by adding an EMA attention mechanism and Focal loss function based on the YOLOv8 framework, addressing the complex background and imbalance between positive and negative samples in the sugarcane dataset. This approach enabled computer vision technology to solve the challenges of sugarcane growth monitoring and disease detection in complex environments (Sun et al., 2024). Some researchers also processed the extracted features during the disease detection stage by introducing a hybrid classifier to obtain better prediction results. This method achieved the highest accuracy in 80% of the learning process (Mangrule and Afreen, 2024). Ong et al. (2023) used visible near infrared spectroscopy (380~1400 nm) in combination with a new wavelength selection method, namely the improved flower pollination algorithm (MFPA), to identify sugarcane diseases. The experimental results show that the simplified SVM model based on MFPA wavelength selection method has the best performance and is superior to the results of other wavelength selection methods, including selectivity ratio, the importance of variables in projection and the baseline method of flower pollination algorithm. Ö rek ç i S ü leyman et al. (Ogrekci et al., 2023). The experimental results show that the accuracy is 92.87%, 93.34% and 87.37% respectively. Ajay Chakravarty et al. (2024) proposed an application based on smart phones, which loads a convolutional neural network model to identify sugarcane plant diseases from images. Kuppusamy et al. (2024b) proposed a new hybrid shift vision converter method for automatic classification of sugarcane leaf diseases. The model integrates the vision converter architecture with the hybrid shift window to effectively capture local and global features. The experimental results show that the hybrid shift converter is superior to the traditional model and achieves a higher accuracy of 98.5% in disease detection. Rajput et al. (2025) proposed a multi-layer Transformer based sugarcane disease classifier (MLTSDC) model to solve this problem. The model uses two levels of classification: the first level identifies the existence of abnormal features, and the second level maps abnormal features to corresponding diseases. The classification accuracy of the proposed model for different diseases affecting sugarcane leaves in the real world reached 98.8%.

In summary, the current literature review reveals a strong interest in the academic community in utilizing deep learning, machine learning, and biosensing technologies to achieve accurate and timely diagnosis of sugarcane diseases. However, precise identification of sugarcane diseases in natural planting environments remains a highly challenging task. This challenge primarily stems from the following aspects:

1. Noise interference in complex environments: Sugarcane plants typically thrive in wild settings, characterized by intricate backgrounds and variable lighting conditions. These factors significantly disrupt the extraction of disease characteristics, rendering existing methods inadequate for accurately identifying disease targets.
2. The Synergistic Challenges of Small Sample Size and Interclass Imbalance: Current research on sugarcane disease recognition predominantly focuses on a limited number of typical diseases (such as rust and yellow leaf diseases), often confined to specific target populations or conditions. These methodologies exhibit suboptimal performance in complex scenarios involving multiple objectives, multiple categories, and class imbalance, making it difficult to meet the demands of practical agricultural production.
3. Inadequate Regional Adaptability: Existing methodologies exhibit significant deficiencies in cross-scenario adaptability, particularly when confronted with diverse crop disease detection tasks. The robustness and generalizability of the models are suboptimal, rendering them challenging to directly apply to disease identification in other crops.

Therefore, this study utilizes public datasets of sugarcane images from various regions, encompassing eight disease categories, including healthy conditions (brown spot disease, eye spot disease, red rot disease, rust disease, yellow leaf disease, mosaic disease, bacterial stripe disease), aiming to construct a more universally applicable and precise sugarcane disease recognition model. This is intended to provide robust technical support for early warning and precise prevention and control of sugarcane diseases. The main contributions are as follows:

1. A trinity improvement framework consisting of “Dynamic Detection Head + Adaptive Label Assignment + Quality Focal Loss” is proposed. Initially, the head of YOLOv8M is modified to DynamicHead, enabling the model to employ attention mechanisms in three dimensions: scale perception, spatial perception, and task perception, significantly enhancing the representational power of the object detection head. Subsequently, the ATSS dynamic label assignment strategy and QFocalLoss loss function are introduced after the Detect module to address issues such as class imbalance, thereby augmenting the model’s feature representation capacity and improving its predictive accuracy and robustness.
2. Verify the model’s generalization capability across crop scenarios. Through the generalization experiments of the ADQ-YOLOv8m model proposed in this study on tomato disease datasets and corn disease datasets, it is confirmed that the proposed model exhibits strong generalization capabilities. The improvement methods outlined in this research can serve as a reference for enhancing crop disease recognition models belonging to the same grass family and sharing similar disease characteristics.

## 2 Materials and methods

### 2.1 Dataset preparation

**Figure 1:**
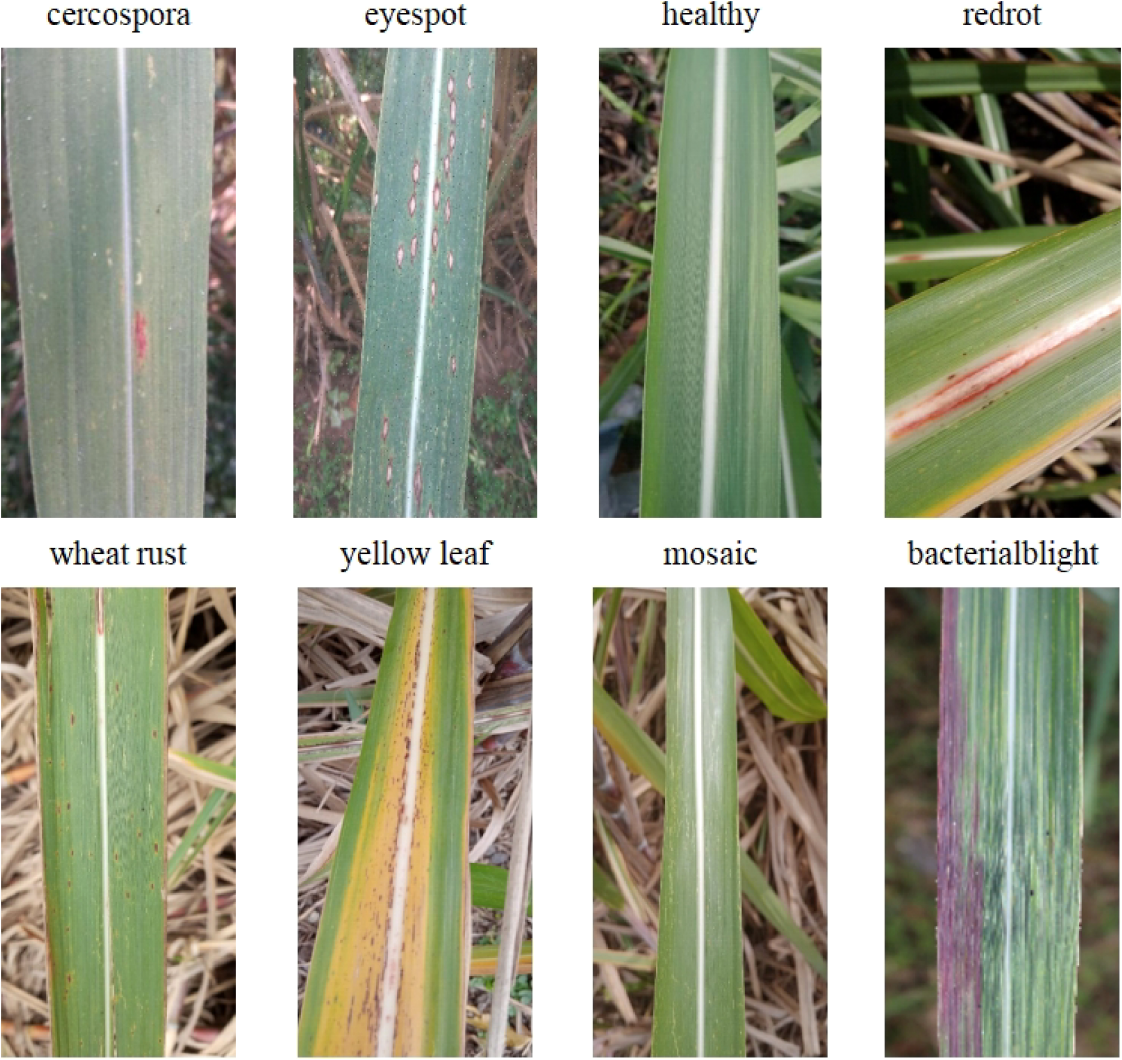
Examples of eight typical categories of sugarcane diseases.

The dataset for this study comprises three datasets: Dataset 1 and Dataset 2 from the Kaggle dataset portal, and a third dataset of sugarcane disease images collected through web scraping. The dataset encompasses eight categories, namely brown spot disease, eye spot disease, healthy, red rot disease, rust disease, yellow leaf disease, mosaic disease, and bacterial stripe disease, with a total of 6,871 images, as illustrated in **Figure 1**. The data set is summarized in **Table 1**.

**Table 1:** Dataset summary.

Dataset	Source	Category	Number of images
Dataset 1	Public dataset: https://www.kaggle.com/datasets/nirmalsankalana/sugarcane-leaf-disease-dataset	5 categories:healthy, mosaic disease, red rot disease, rust disease, and yellow leaf disease	2569
Dataset 2	Public dataset: https://www.kaggle.com/datasets/akilesh253/sugarcane-plant-diseases-dataset	3 categories:bacterialblight, mosaic leaf disease and yellow leaf	1564
Dataset 3	Network crawling	3 categories:cercospora, eye spot disease, mosaic disease	2738

Dataset 1: A manually collected dataset of sugarcane leaf disease images. It primarily comprises five major categories: healthy, mosaic disease, red rot disease, rust disease, and yellow leaf disease. The dataset has been captured using smartphones of various configurations to maintain diversity. It encompasses a total of 2569 images, encompassing all categories. The database has been collected in the state of Maharashtra, India. The database is balanced and exhibits a good diversity. The image sizes are not uniform, as they originate from various capture devices. All images are in RGB format. This study utilized the entire set of images from Dataset 1. Source: https://www.kaggle.com/datasets/nirmalsankalana/sugarcane-leaf-disease-dataset.
Dataset 2: This dataset comprises 19,926 images of sugarcane leaves, categorized into six distinct classes. Each class represents a specific condition of the sugarcane leaves, encompassing healthy specimens as well as varieties afflicted with various diseases. The dataset has undergone data augmentation techniques, including rotation, flipping, scaling, resizing, and cropping, to enhance model training. The detailed classification of the dataset is as follows: 4,800 images of bacterial wilt disease, 3,132 images of healthy leaves, 2,772 images of mosaic disease, 3,108 images of red rot disease, 3,084 images of rust disease, and 3,030 images of yellow disease. All images are in PNG format. In order to ensure the pertinence and data balance of the experiment, we selected three categories of bacterial wilt, mosaic leaf disease and yellow disease, and selected a total of 1564 original images according to the number of samples, image quality and representativeness of the categories, which will be uniformly enhanced by me later. The selection criteria mainly include: ① ensure that the number of samples in each category is statistically representative in training; ② High image definition without serious noise or occlusion; ③ It covers the diversity of different leaf growth states and disease manifestations. Source: https://www.kaggle.com/datasets/akilesh253/sugarcane-plant-diseases-dataset.
Dataset 3: A web crawler was designed in Python, targeting major search engines, specialized agricultural image databases, and publicly available plant disease dataset sharing platforms. A total of 2738 images, encompassing brown spot disease, eye spot disease, mosaic disease, and additional categories, were retrieved. The crawled images are subject to a strict data cleaning process, including: ① de duplication: automatic removal of duplicate or highly similar images using perceptual hash method; ② Definition screening: eliminate blurred, overexposed or incompletely cropped images; ③ Consistency of annotation: all images are annotated by two agronomic experts. If there are differences, the third expert will arbitrate to ensure the accuracy and consistency of category annotation.

### 2.2 Data enhancement

Gamma correction is a nonlinear transformation used to correct brightness deviations and contrast in images, helping to make the brightness distribution more uniform and improve visual quality. Hue adjustment can change the overall color tendency of an image, making it more aligned with detection requirements. In pest and disease detection, images are sourced from different environments and conditions.

**Figure 2:**
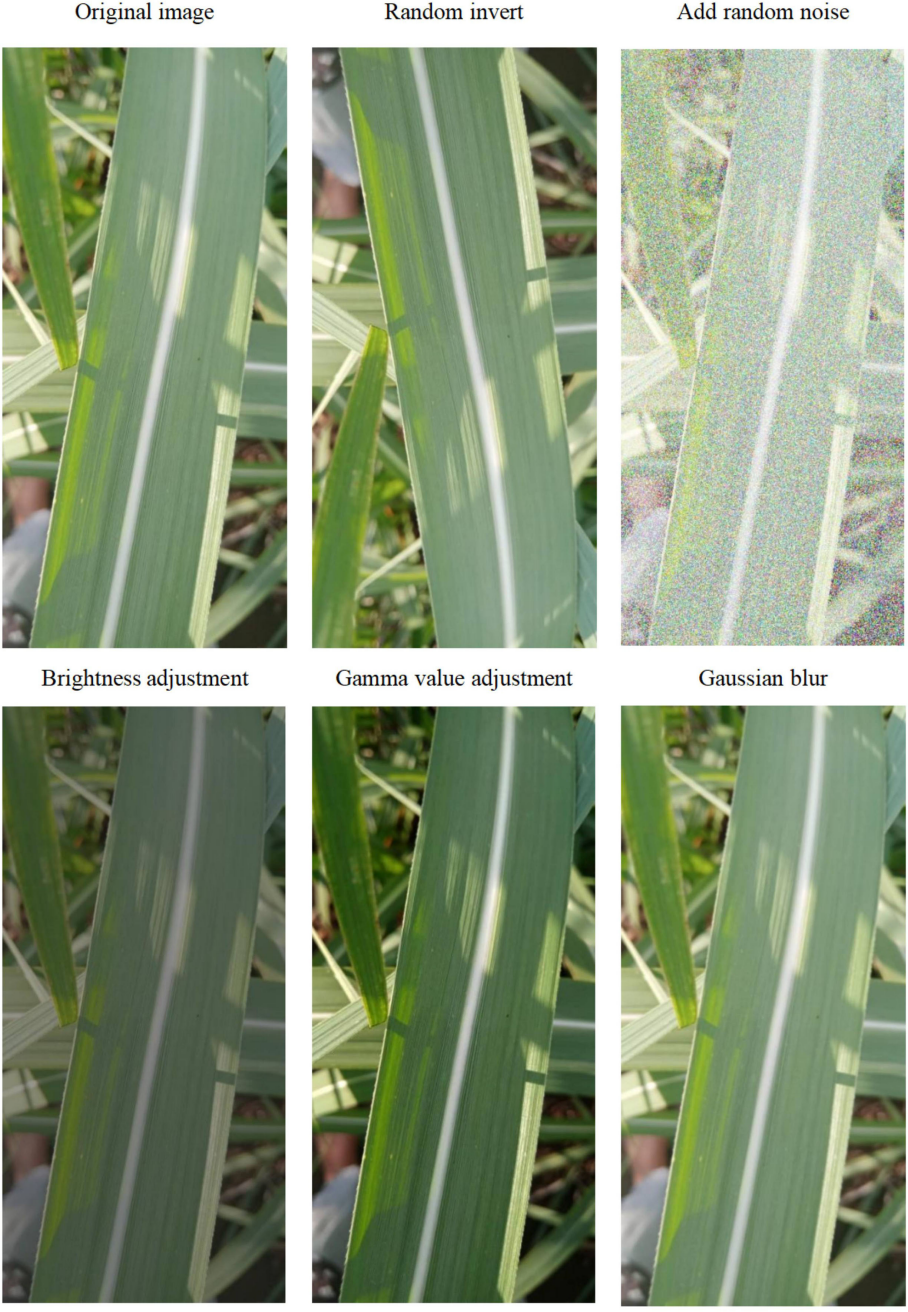
Illustration of data augmentation methods.

By adjusting the image’s Gamma value and hue, the brightness and contrast of the image can be optimized, enhancing image quality to adapt to different environmental conditions, thereby improving detection accuracy. Building a sugarcane disease detection model requires establishing an image dataset based on image data and performing data augmentation on this dataset. For this experiment, preprocessing methods such as random horizontal/vertical flipping, adjusting brightness/contrast, gamma value, and adding random noise were used to expand the dataset threefold, forming a data resource library, as shown in **Figure 2** and **Table 2**. After expansion, there were a total of 18,810 images. The LabelImg software was used to annotate the target for these 18,810 sugarcane leaf disease images. The dataset was then divided into training, testing, and validation sets in an 8:2 ratio, forming a complete dataset for training and testing.

**Table 2:** Label distribution of sugarcane disease image dataset.

Disease name	Category label quantity	Enhanced label quantity	Total quantity
cercospora	7189	21567	28756
eyespot	344	1032	1376
healthy	1247	3741	4988
redrot	275	825	1100
wheat rust	778	2334	3112
yellow leaf	1512	4536	6048
mosaic	2258	6774	9032
bacterialblight	2511	7533	10044
Total	16114	48342	64456

### 2.3 Technology roadmap

**Figure 3:**
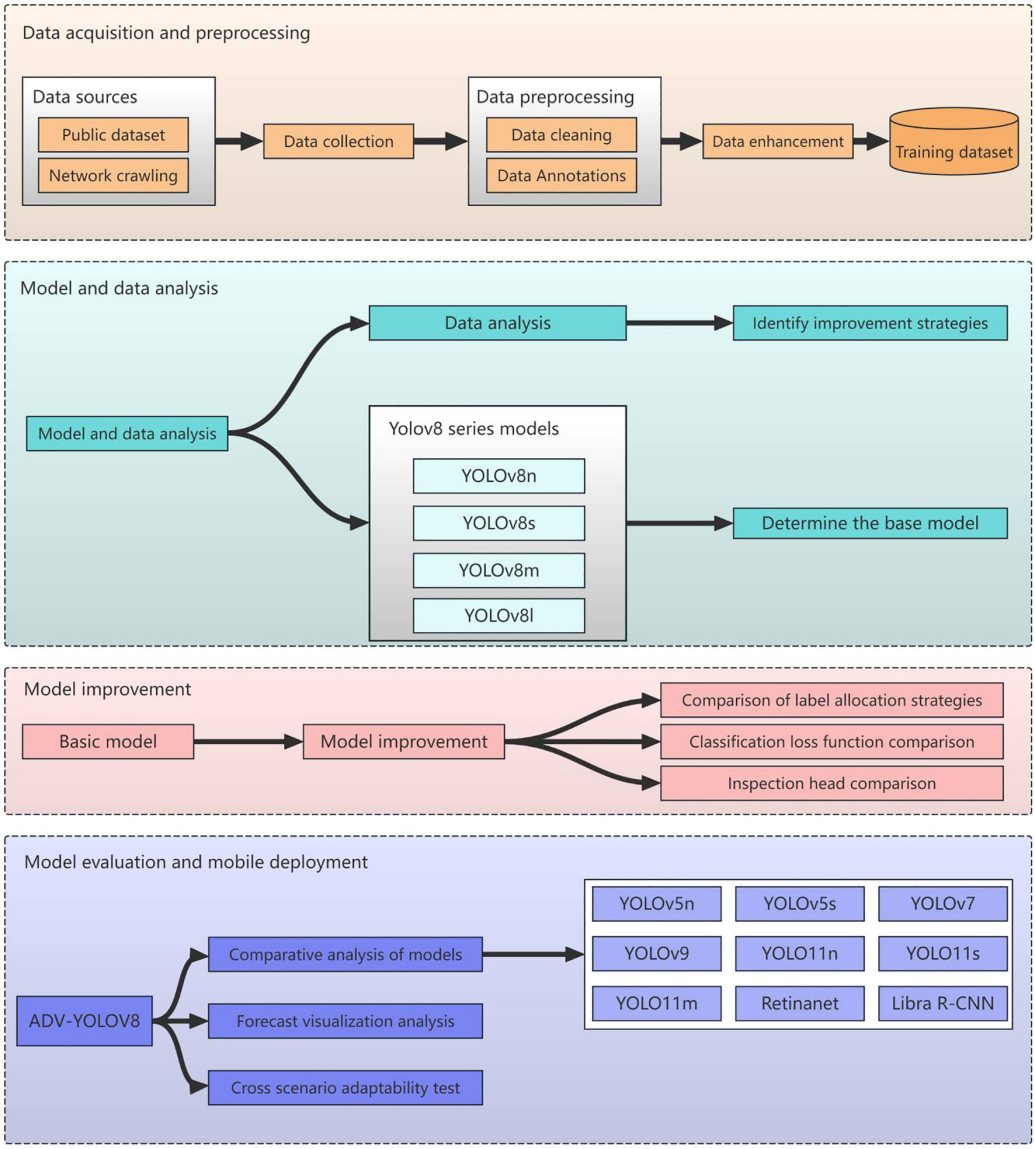
Technical roadmap for sugarcane disease detection.

The technical approach outlined in this paper is depicted in **Figure 3**.

#### 2.3.1 Data acquisition and data preprocessing

Initially, we endeavor to gather a comprehensive array of sugarcane disease categories from both public datasets and the internet. Following this, we embark on data cleansing and employ a range of data augmentation techniques, including rotation, flipping, and adjustments to brightness, contrast, and saturation, to augment the dataset and enhance the model’s generalization capabilities across diverse scenarios. The preprocessed and augmented data are then amalgamated into a comprehensive training dataset, which is subsequently split into training and validation sets to pave the way for model training.

#### 2.3.2 Modeling and data analytics

Conduct a comprehensive analysis of the training dataset, encompassing the quantity of instances across various categories, the spatial distribution of bounding boxes predicted by the model within the images, and the positional distribution of these predicted bounding boxes. This analysis aims to comprehend the characteristics of the data, thereby furnishing a solid foundation for model selection and enhancement. Based on the insights gleaned from the data analysis, strategize for model improvement, such as implementing specific sampling techniques or adjusting the loss function to address category imbalance issues.

Conduct a comprehensive study on the YOLOv8 series of models (YOLOv8n, YOLOv8s, YOLOv8m, YOLOv8l), analyzing the structural characteristics, parameter counts, computational complexity, and performance on various datasets. Aligning with project requirements and data characteristics, a holistic evaluation is conducted in terms of accuracy, speed, and model size to determine the optimal foundational model.

#### 2.3.3 Model training and refinement optimization

The YOLOv8s model, which strikes a balance between speed and accuracy, has been selected for enhancements in label assignment strategy, loss function, and detection head. These improvements aim to address issues such as class imbalance, bolster the model’s feature representation capabilities, and elevate its predictive accuracy and robustness.

#### 2.3.4 Model evaluation and generalization studies

Utilizing standard metrics such as mAP, Precision, Recall, and F1-score, a comprehensive evaluation was conducted on the enhanced model, ADV-YOLOv8, to compare its performance with that of the baseline model and nine other leading object detection models, including YOLOv5n, YOLOv7, YOLOv9, YOLOv11n, YOLOv11s, YOLOv11m, and Retinanet, thereby verifying the model’s performance enhancement. By visualizing the model’s predictions on images, the detection outcomes for various targets were displayed, providing an intuitive assessment of the model’s detection efficacy and feature learning capabilities for further optimization. Simultaneously, the evaluated and optimized ADV-YOLOv8 model underwent cross-scenario adaptability testing to analyze its effectiveness and stability in different crop disease scenarios.

### 2.4 Model overview

#### 2.4.1 YOLOv5

YOLOv5, released by Glenn Jocher in 2020, stands out notably for its incorporation of the Focus and CSPDarknet-53 structures within its backbone network. The Focus structure, a pivotal component of YOLOv5, is designed to extract high-resolution features. It employs a lightweight convolutional operation, enabling the model to maintain a large receptive field while reducing computational demands. CSP (Cross Stage Partial) Darknet-53, the backbone network structure in YOLOv5, introduces the concept of cross-stage partial connections. By dividing the feature map into two parts along the channel dimension, it maintains high feature representation capabilities, thereby enhancing the accuracy and speed of object detection. YOLOv5 is available in five versions: YOLOv5n, YOLOv5s, YOLOv5m, YOLOv5l, and YOLOv5x. Notably, YOLOv5n boasts the shallowest depth and the narrowest feature map width within the series (Yao et al., 2021).

#### 2.4.2 YOLOv7

YOLOv7 has achieved a dual breakthrough in detection speed and accuracy within the field of object detection. Its core innovations include the Extended Efficient Layer Aggregation Network (E-ELAN) and the trainable “Bag of Freebies” training strategy. E-ELAN enhances gradient propagation efficiency through grouped convolution and feature rearrangement, while dynamic label assignment optimizes the target matching issue across multiple output layers. YOLOv7 employs model reparameterization technology, which consolidates the multi-branch structure during training into a single branch, thereby reducing the number of parameters and computational load during inference (Nikarika et al., 2024).

#### 2.4.3 YOLOv8

**Figure 4:**
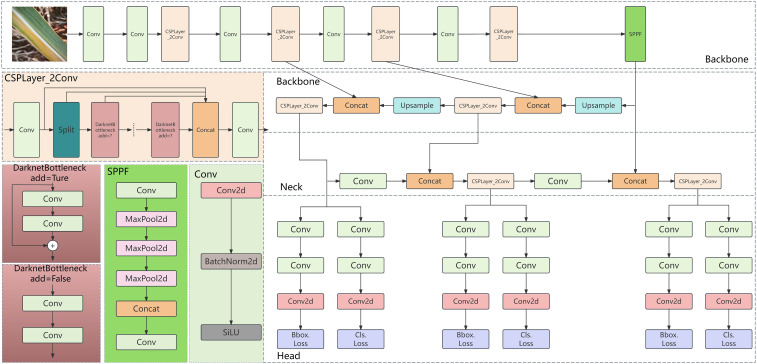
Overview of the YOLOv8 network architecture.

YOLOv8, an object detection model open-sourced by ultralytics in January 2023, is an updated version based on YOLOv5. It maintains high accuracy and enhances inference speed while further reducing model weight. YOLOv8 is available in various versions, with model parameters ranging from YOLOv8n to YOLOv8x, representing increasing sizes. Yolov8n is an extremely lightweight model with high computational efficiency, but its feature extraction ability is weak, so it is difficult to detect crops with complex background; Yolov8s has both efficiency and performance, but it is insufficient to detect small targets and rare diseases; Yolov8m has both high precision and computational cost, and has strong feature extraction ability; Yolov8l has high detection performance, but it has high computational complexity and low efficiency, which is not conducive to the environment with limited resources; Yolov8x has the strongest performance and the best feature expression ability, but it is difficult to apply because of its high computational cost and complex model; Compared with yoov8n and yoov8s, yoov8m can significantly improve performance and efficiency, and can cope with small targets and unbalanced data. Compared with yoov8l and yoov8x, yoov8m has similar performance, but significantly reduces the computational cost, which is more suitable for agricultural applications. The model structure primarily consists of four components: the input, backbone network, neck network, and detection head (Yaseen, 2024). The network architecture is illustrated in **Figure 4**.

#### 2.4.4 YOLOv9

YOLOv9, a newer iteration of the YOLO series, has undergone further refinement in its model architecture and training strategies. The backbone network incorporates an enhanced C2F module, replacing the C3 module from YOLOv8. This integration utilizes depthwise separable convolution to minimize computational overhead and employs the α-CIoU loss function to expedite convergence and elevate localization precision. Moreover, YOLOv9 introduces the Dynamic Feature Pyramid Network (DFPN), bolstering the detection capabilities across multiple scales, particularly excelling in intricate backgrounds and scenarios involving small targets. Additionally, YOLOv9 facilitates anchor-free detection, simplifying the model structure and diminishing reliance on hyperparameters (Wang et al., 2024).

#### 2.4.5 RetinaNet

RetinaNet, a single-stage object detection model, was introduced by Facebook AI Research (FAIR) in 2017. It employs Focal Loss as its loss function, effectively addressing the challenge of single-stage detectors dealing with extreme imbalances in foreground and background class data (Wang et al., 2019). RetinaNet integrates the Feature Pyramid Network (FPN) with the anchor box mechanism, utilizing ResNet as the backbone network to extract multi-scale features and generate pyramid feature maps through the FPN, thereby enhancing the detection capability for targets of varying sizes. Compared to traditional two-stage detectors such as Faster R-CNN, RetinaNet offers both high detection accuracy and inference speed. On the COCO dataset, it achieves accuracy comparable to that of two-stage detectors while maintaining the efficiency of a single-stage model.

#### 2.4.6 Libra R-CNN

Libra R-CNN is an enhanced object detection model, built upon the Faster R-CNN architecture, specifically designed to address the issue of sample imbalance and enhance detection accuracy. Its core philosophy lies in optimizing the training process through the integration of three strategies: Balanced Sampling, Balanced L1 Loss, and IoU-balanced Sampling. This approach mitigates the impact of high-quality target factors on lower-quality ones, thereby improving detection precision (Pang et al., 2019).

In Libra R-CNN, the Focal Sampling Assignment (FSA) mechanism is employed for balanced sampling, mitigating the imbalance between positive and negative samples during the Region Proposal Network (RPN) phase. The balanced L1 loss equalizes the impact of difficult and easy samples on the loss by adjusting gradient contributions. Additionally, the IoU balancing mechanism optimizes the Region of Interest (RoI) selection process, enhancing the utilization of high IoU samples. These enhancements position Libra R-CNN to outperform the standard Faster R-CNN on datasets such as COCO, particularly in detecting small targets and targets within the low IoU range, where significant improvements are observed.

### 2.5 Dynamic Tagging Strategy System

ATSS (Adaptive Training Sample Selection) is a dynamic label assignment strategy based on prior information, specifically tailored for object detection tasks. It enhances model performance and efficiency by adaptively selecting positive and negative samples (Zhang et al., 2022). The underlying principle involves generating a series of candidate samples at each feature level based on preset anchor boxes. For each ground truth (GT) label, the Intersection Over Union (IoU) values between all candidate samples and the GT are computed. Subsequently, thresholds for positive and negative samples are dynamically set based on the mean and standard deviation of the IoU values. Candidate samples are then categorized as positive or negative according to these thresholds. This approach effectively reduces interference from redundant samples by dynamically selecting high-quality samples, thereby enhancing detection performance.

### 2.6 Dynamic Detection Head

The DynamicHead architecture primarily encompasses three distinct attention mechanisms, the characteristics of its structure and the functions of each attention mechanism are as follows (Dai et al., 2021):

Overall Architecture: Initially, various backbone networks are employed to extract feature pyramids, which are then adjusted to the same scale, forming a 3-dimensional tensor that is subsequently input into the DynamicHead. Subsequently, multiple DyHead blocks, encompassing scale-aware, spatial-aware, and task-aware attention, are sequentially stacked. Their outputs can be utilized for diverse tasks and representations in object detection, such as classification, center/box regression, among others.

Scale-aware Attention: Deployed exclusively at the feature hierarchy dimension, it enhances features at appropriate levels based on object scales by learning the relative importance of different semantic hierarchies. As shown in Equation (1):


(1) $$\pi_{\text{L}}(F)\cdot F=\sigma (f(\frac{1}{\text{SC}}\sum_{\text{S},\text{C}}F))\cdot F$$

In this context, f(·) represents a linear function approximated by a 1×1 convolutional layer, while $\sigma (\text{x})=max(0,min(1,\frac{\text{x}+1}{2}))$ denotes the hard sigmoid function.

Where $\pi_{\text{L}}(F)$ is the scale aware attention function, which is used to weight the attention of the input feature F in the scale dimension, F is the input feature pyramid tensor, f (·) is a linear function approximated by 1 × 1 convolution layer, SC is the product of the spatial size of the feature and the number of channels, is the global summation of the feature in the spatial dimension (s) and the channel dimension (c), and aggregates the multi-scale feature information, σ(·) is a hard sigmoid function. The formula is $\sigma (\text{x})=max(0,min(1,\frac{\text{x}+1}{2}))$ is a hard sigmoid function.

Spatial-aware Attention: Deployed in the spatial dimension (height × width), it first learns sparse attention through deformable convolution, and then aggregates cross-level features at the same spatial location to focus on discriminative regions that consistently exist across spatial locations and feature levels. As shown in Equation (2):


(2) $$\begin{array}{c} \pi_{S}(F)\cdot F=\frac{1}{L}\sum_{l=1}^{L}\sum_{k=1}^{K}w_{l,k}\cdot F(l;p_{k}+\Delta p_{k};c)\cdot \Delta m_{k} \end{array}$$

$\pi_{\text{S}}(F)$ denotes the spatial-aware attention function, where L represents the number of levels in the feature pyramid, K indicates the number of sampled key points per spatial location, ${\text{w}}_{\text{l},\text{k}}$ denotes the weighting coefficient at the k-th sampled position in the I-th level features, and $F(\text{l};{\text{p}}_{\text{k}}+{\text{Δp}}_{\text{k}};\text{c})$ represents the feature value at position ${\text{p}}_{\text{k}}+{\text{Δp}}_{\text{k}}$ and channel c in the I-th level features. Specifically: ${\text{p}}_{\text{k}}$ refers to the pre-defined initial sampling position coordinates, ${\text{Δp}}_{\text{k}}$ represents the self-learned spatial offset for dynamic adjustment of sampling positions, focusing on discriminative regions, c indicates the feature channel index, ${\text{Δm}}_{\text{k}}$ corresponds to the importance scalar at the k-th sampled position, and $\frac{1}{L}\sum_{\text{l}=1}^{\text{L}}$ performs average aggregation across all levels to integrate multi-scale spatial information.

Task-aware Attention: Deployed on the channel, it dynamically switches feature channels based on the responses of different convolutional kernels for objects, supporting various tasks such as classification, bounding box regression, center/keypoint learning, etc. As shown in Equation (3):


(3) $$\begin{array}{c} \pi_{C}(F)\cdot F=max(\alpha^{1}(F)\cdot F_{c}+\beta^{1}(F),\alpha^{2}(F)\cdot F_{c}+\beta^{2}(F)) \end{array}$$

Where $\pi_{C}$ is the task perceived attention function, and $F_{c}$ is the feature slice of the c-th channel in feature F, ${\left[\alpha^{1},\alpha^{2},\beta^{1},\beta^{2}\right]}^{T}=\theta (\cdot )$ , Where $\alpha^{1},\alpha^{2}$ is the channel weight coefficient, $\beta^{1},\beta^{2}$ is the offset term, θ(·) is a network composed of global average pooling, two full connection layers, normalization layer and shift sigmoid function, which is used to learn task related attention parameters, dynamically adapt to the needs of classification or regression tasks, and max(·) is the maximum value of the weighted results of the two channels to strengthen the channel characteristics that are more important to the task.

### 2.7 QFocalLoss loss function

Focal Loss addresses the class imbalance issue by introducing a modulation coefficient ${\left(1-p_{t}\right)}^{\gamma }$ and a balancing parameter α, which enables the model to focus more on difficult-to-classify samples. However, its reliance on discrete labels (such as 0/1 labels indicating the presence or absence of an object in the target) precludes it from handling label smoothing or probability mass distributions (such as continuous labels in IoU-aware classification tasks)

QFocal Loss (Quality Focal Loss) is an extended version of Focal Loss, designed to address the limitation of traditional Focal Loss, which only supports discrete labels (0/1 binary labels). It is particularly suitable for scenarios requiring label smoothing or probability distribution handling (Li et al., 2020).

The modulation coefficient of traditional Focal Loss is ${\left(1-p_{t}\right)}^{\gamma }$ , where $p_{t}$ represents the degree of alignment between the predicted probability and the true label. QFocal Loss, however, substitutes it with $|y-\sigma {|}^{\gamma }$ (where y denotes the true label and σ represents the predicted probability), thereby facilitating continuous labels.

As shown in Equation (4):


(4) $$\begin{array}{c} QFL(\text{σ})=-{\text{α}}_{t}\cdot |\text{y}-\text{σ}{|}^{\text{γ}}\cdot [(1-\text{y})\text{log}(1-\text{σ})+\text{ylog}(\text{σ})] \end{array}$$

$\alpha_{t}$ : The weight balancing positive and negative samples (for instance: $\;\alpha_{t}=y\cdot \alpha +(1-y)\cdot (1-\alpha )$ ); $|y-\sigma {|}^{\gamma }$ : Dynamically adjust the weights of difficult and easy samples, with higher weights assigned when there is a significant discrepancy between the predicted and actual labels.

### 2.8 Construction of the ADQ-YOLOv8m model

**Figure 5:**
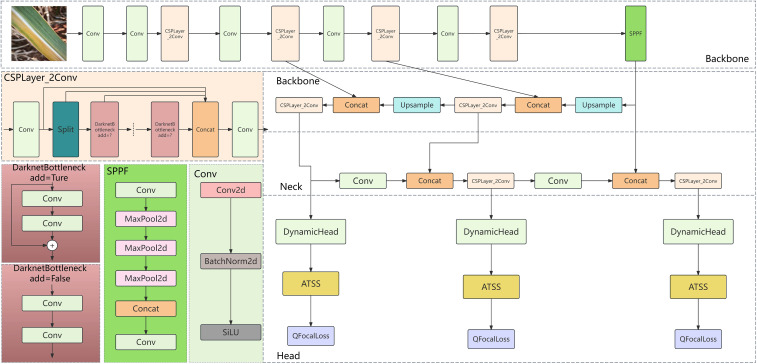
Schematic diagram of the ADQ-YOLOv8m model architecture.

Addressing the imbalances in instance quantity and foreground-background categories within the sugarcane disease dataset of this study, we employed YOLOv8M as the foundational model and implemented three enhancements to the network:

1. Incorporate a Dynamic Head detection system into the Head section;
2. ATSS necessitates the dynamic allocation of positive and negative sample labels based on the model’s predictive outcomes (preliminary detection results). The choice of these labels has a direct impact on the computation of the loss function. Consequently, the dynamic label assignment strategy for ATSS ought to be integrated post the Detect module within the Head section, prior to the loss computation process;
3. The model is optimized using the QFocal loss function and the IoU loss function, with the improvement locations depicted in **Figure 5**.

### 2.9 Evaluation metrics

mAP_0.5: mAP_0.5 represents the average of the mean accuracy across all categories when the IoU threshold is set at 0.5. The mean Average Precision (mAP) is a metric that quantifies the average precision (AP) across all detected target categories. The average precision (AP) is used to assess the efficacy of detection for a specific category, specifically, the recognition accuracy in the detection of sugarcane diseases. Meanwhile, the mean mAP serves as a measure of detection performance across multiple categories, reflecting the overall recognition effectiveness for all types of sugarcane diseases. As shown in Equation (5):


(5) $$\begin{array}{c} AP=\sum_{i=0}^{n-1}\left(r_{i+1}-r_{i}\right) \end{array}$$

In this context, r1, r2…rn represent the Recall values corresponding to the first interpolation point of the Precison interpolation segments, arranged in ascending order. Pi denotes the precision of the ith detection. As shown in Equation (6):


(6) $$\begin{array}{c} mAP=\frac{\sum AP}{N(classes)} \end{array}$$

Precision: The ratio of the number of instances of a particular feature detected to the total number of features detected, serving as an indicator of the model’s accuracy in identifying the target. As shown in Equation (7):


(7) $$\begin{array}{c} Precision=\frac{True\;Positive}{True\;Positive+False\;Positive}=\frac{TP}{TP+FP} \end{array}$$

In this context, True Positive represents the positive samples that the network model correctly identifies as disease, False Positive denotes the negative samples that the network model incorrectly identifies as disease, and False Negative signifies the positive samples that the network model fails to detect as disease, essentially representing false negatives.

Recall: The ratio of the number of features detected as belonging to a specific class to the total number of features of that class in the dataset. It measures the completeness of the model’s detection system. As shown in Equation (8):


(8) $$\begin{array}{c} Recall=\frac{True\;Positive}{True\;Positive+False\;Negative}=\frac{TP}{TP+FN} \end{array}$$

In this context, True Positive refers to the positive samples that the network model correctly identifies as disease-affected, while False Positive denotes the negative samples that the model erroneously classifies as diseased. False Negative, on the other hand, signifies the positive samples that the model fails to detect as disease-affected, essentially representing a false negative.

F1: The harmonic mean of precision and recall. F1 integrates the considerations of both precision and recall. As shown in Equation (9):


(9) $$\begin{array}{c} F=\frac{2PR}{\text{α}(P+R)} \end{array}$$

In this context, P denotes Precision, R stands for Recall, and α represents a weighting factor. When α equals 1, it signifies that the precision and recall are given equal weight, resulting in F being equivalent to F1. As shown in Equation (10):


(10) $$\begin{array}{c} F1=\frac{2PR}{P+R} \end{array}$$

Generally, a higher F1 score indicates a more effective model.

Gradient: In machine learning models, the gradient refers to the partial derivative of the objective function with respect to the model parameters. It signifies the rate of change and direction of the objective function at the current point.

FLOPs: A metric used to assess the computational complexity of a model. FLOPs, which stands for floating-point operations, represents the number of floating-point operations required for a single forward pass through the model. In assessing computational complexity, a higher FLOPS indicates greater computational cost and longer inference time. The convolution layer is shown in Equation (11):


(11) $$\begin{array}{c} FLOPs=\frac{H_{out}W_{out}(C_{in}(2K^{2}-1)}{g+1} \end{array}$$

Hout and Wout respectively denote the height and width of the output from the convolutional layer, Cin represents the number of input channels, K stands for the size of the convolution kernel, Cout signifies the number of output channels, g is the number of groups in grouped convolution, and the addition of +1 accounts for convolutions with bias. The fully connected layer is shown in Equation (12):


(12) $$\begin{array}{c} FLOPs=((2C_{in}-1)+1)C_{out}=2C_{in}C_{out} \end{array}$$

## 3 Experimental results and analysis

### 3.1 Experimental apparatus

The hardware configuration for this experiment includes a central processing unit (CPU) of 16 vCPU Intel(R) Xeon(R) Gold 6430, with 120GB of operational memory, a graphics processing unit (GPU) of NVIDIA GeForce RTX 4090 (24GB), and a 1TB solid-state drive. The software system is based on the Ubuntu 20.04.5 LTS operating system. All programs are executed under the Python 3.8.10 environment and the deep learning framework Pytorch 2.0.0, utilizing the NVIDIA CUDA 11.8 parallel computing driver to accelerate training. The model training parameters are set as follows: image size 640*640, batch_size 64, epochs 500, optimizer SGD, label smoothness 0.5, initial learning rate 0.01, final learning rate 0.0001, optimizer momentum 0.937, and optimizer weight decay 0.0005.

### 3.2 Selection of fundamental models for sugarcane disease recognition

Comparative experiments were conducted on the sugarcane disease dataset using YOLOv8n, YOLOv8s, YOLOv8m, and YOLOv8l, respectively. Metrics such as accuracy, recall, mAP_0.5, F1 score, depth, parameter count, gradient, and FLOPS (G) were selected to assess the training accuracy and loss function values of the models on the test set, aiming to identify the optimal base model.

The experimental results, as presented in **Table 3**, indicate a significant enhancement in training performance with the increase in the number of model parameters and complexity. Specifically, the mAP50 score rose from 65.70% for YOLOv8n to 88.70% for YOLOv8l. While the YOLOv8l model boasts the highest training performance, its improvement in model performance is relatively modest, increasing by only 3.2% compared to 8m. In terms of precision, recall, mAP50, mAP50-95, and F1 score, YOLOv8m slightly trails behind 8l, with values of 85.2%, 81.4%, 85.90%, 74.20%, and 84%, respectively. However, YOLOv8m strikes a good balance between precision and recall, offering both accurate and comprehensive disease detection. Its comprehensive performance is robust, demonstrating superior recognition rates and generalization capabilities in sugarcane disease detection tasks. Therefore, YOLOv8m is selected as the foundational model for this study.

**Table 3:** Performance analysis table of four sugarcane disease detection models utilizing YOLOv8.

Model	Precision	Recall	mAP50	mAP50-95	F1	Depths	Total parameters	FLOPS (G)
YOLOv8n	65.70%	61.00%	65.70%	35.90%	63.00%	225	3012408	8.2
YOLOv8s	73.60%	71.90%	76.40%	47.30%	73.00%	225	11138696	28.7
YOLOv8m	85.20%	81.40%	85.90%	74.20%	84.00%	295	25860952	79.1
YOLOv8l	90.00%	82.50%	88.70%	79.10%	86.00%	365	43636008	165.4

**Figure 6:**
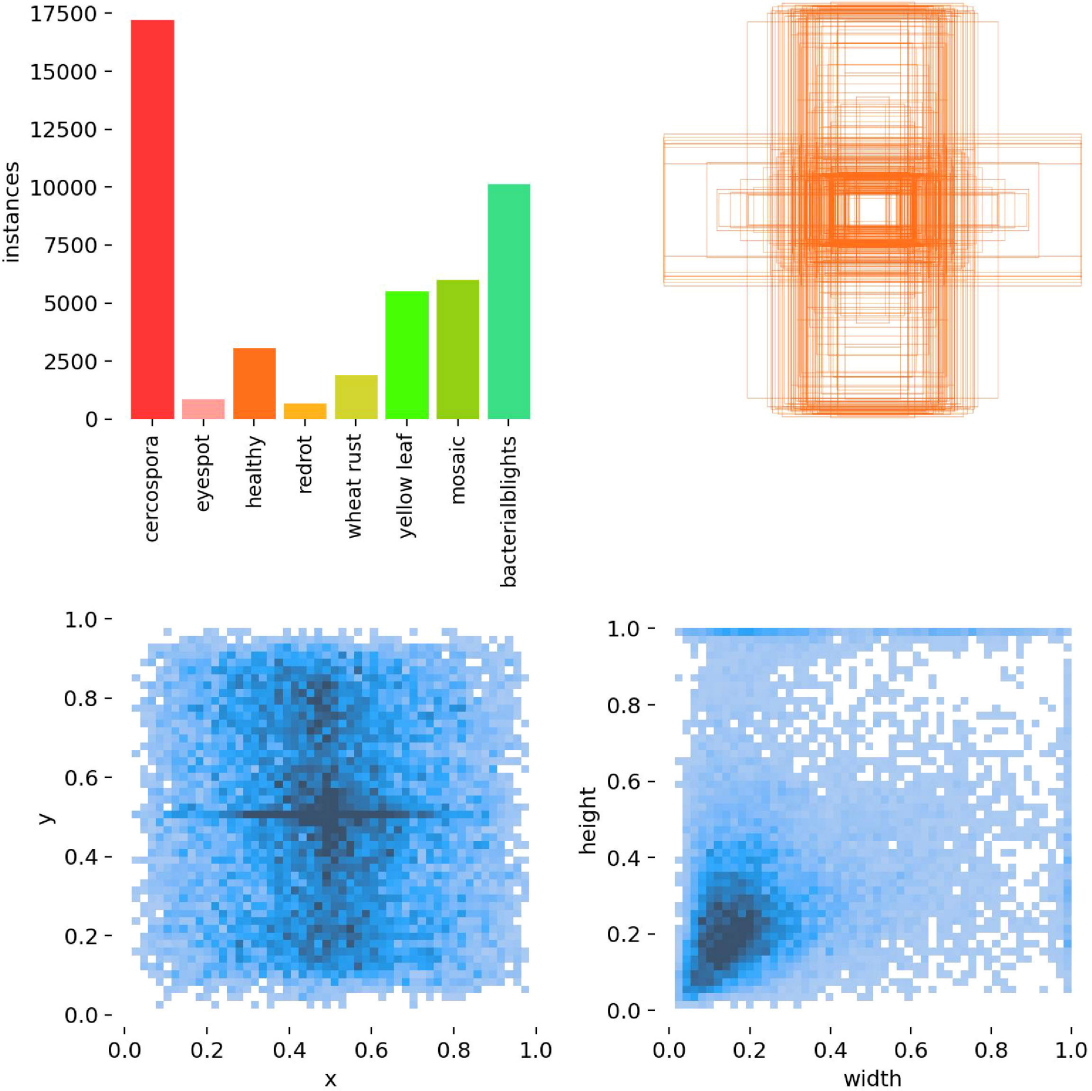
Analysis of the sugarcane disease dataset distribution. Top left: Number of samples in each category. Top right: Distribution of ground-truth bounding box positions. Bottom left: Distribution of predicted bounding box positions. Bottom right: Distribution of predicted bounding box sizes (width and height).

**Figure 6** illustrates the distribution of the dataset in this study. The composite chart, as evident from the figure, presents the distribution characteristics of the sample data from multiple perspectives through various sub-charts. The dataset exhibits an imbalance in the number of instances across different categories, and the samples are densely distributed in space, with certain intersections and overlaps. Consequently, subsequent model improvements will primarily address this issue.

### 3.3 Enhancing strategy selection

#### 3.3.1 Comparison of dynamic label allocation strategies

ATSS is an adaptive strategy for positive and negative sample allocation, employed in label assignment for object detection. It operates by identifying the k closest candidate anchor boxes to the Ground Truth (GT) box at each feature level. Subsequently, it computes the Intersection Over Union (IoU) between these candidate boxes and the GT, and determines the IoU threshold by calculating the mean and standard deviation of these IoUs. Boxes with an IoU exceeding this threshold are selected as the final positive samples.

Dynamic ATSS represents an enhanced label assignment strategy, building upon ATSS and incorporating both the Intersection over Union (IoU) of prediction outcomes and the IoU of anchor points to more precisely select positive and negative samples. During the initial stages of training, due to the inaccuracies in prediction results, it primarily relies on the IoU of anchor points as the primary criterion for label definition. As training progresses, the prediction outcomes increasingly dominate the combined IoU, thereby guiding the label assignment during the training phase (Zhang et al., 2022b).

During the training phase of the selected YOLOv8m model’s detection head, two distinct label assignment strategies were integrated to assess their comparative performance.

**Table 4** shows the performance comparison of the two tag allocation strategies. Yoov8m ATSs and yoov8m are better than yoov8m dynamic ATSs in accuracy, recall rate, map50, map50–95 and F1 score. Yoov8m ATSs performs best, indicating that they perform best in prediction accuracy and generalization ability; The introduction of dynamic ATSs reduces the performance of the original model, indicating that the dynamic adjustment strategy may introduce instability. By comparing the performance indicators of the three models, the accuracy of yoov8m ATSs is 88.10%, the recall rate is 79.80%, the map50 is 87.70%, the map50–95 is 72.10%, and the F1 score is 84.00%, which is better than yoov8m and yoov8m dynamic ATSs in accuracy and map50. It can be seen that the ATSs strategy significantly improves the detection performance and generalization ability of the model in complex scenes. This improved strategy solves the limitations of the traditional fixed threshold method by optimizing the label allocation mechanism, and shows stronger generalization ability in complex scenes. Therefore, it is reasonable and effective to choose ATSs as the improvement strategy.

**Table 4:** Comparison of the performance of two label allocation strategies.

Model	Precision	Recall	mAP50	mAP50-95	F1
YOLOv8m	85.20%	81.40%	85.90%	74.20%	84.00%
YOLOv8m-Dynamic ATSS	83.90%	80.00%	85.30%	65.60%	82.00%
YOLOv8m-ATSS	88.10%	79.80%	87.70%	72.10%	84.00%

#### 3.3.2 Selection of detection head

In the task of object detection, the detection head serves as a pivotal component of the model, directly impacting its ability to locate and classify targets. To enhance the performance of the sugarcane disease recognition model, we intend to employ a comparative analysis of various detection head structures, including DynamicHead, ShareSepHead, ImplicitHead, LiteShiftHead, and TransHead. DynamicHead is capable of adaptively allocating attention, ShareSepHead facilitates the sharing of feature extraction parameters to reduce computational demands, ImplicitHead utilizes implicit representations to model target features, LiteShiftHead employs lightweight shift operations to minimize computational complexity, and TransHead incorporates a Transformer structure to bolster global feature modeling capabilities. Consequently, by replacing the original detection head of YOLOv8-ATSS with the aforementioned five detection head structures, we have constructed five distinct model variants. By comparing the performance of these detection heads in the task of sugarcane disease recognition, we aim to select or design a detection head structure more suitable for the task, thereby enhancing the model’s detection accuracy and efficiency.

**Table 5** presents a comparative analysis of the recognition performance of five detection heads, revealing distinct differences in their capabilities for sugarcane disease identification. Among them, DynamicHead exhibits the best performance in terms of recall, mAP50, mAP50-95, and F1 scores, with values of 84.30%, 88.40%, 77.20%, and 86.00%, respectively. This indicates that it achieves an optimal balance between precision and recall, accurately detecting disease targets while minimizing the omission of disease instances. In terms of precision, ImplicitHead and LiteShiftHead demonstrate the best performance, with values of 90.30% and 90.40%. Although dynamiehead ranks fourth in accuracy, which is lower than 90.30% of implicithead, 90.40% of liteshifthead and 89.90% of transhead, it performs well in the other four indicators, which are 84.30%, 88.40%, 77.20% and 86.00% respectively, indicating that it can detect disease targets more comprehensively and reduce omissions, which is suitable for sugarcane disease recognition in complex scenes. Therefore, based on a comprehensive comparison of the performance metrics of the five detection heads, we conclude that DynamicHead exhibits the most comprehensive and excellent performance in sugarcane disease identification, making it the optimal choice among the five detection heads.

**Table 5:** Comparison of recognition performance among five different detection heads.

Model	Precision	Recall	mAP50	mAP50-95	F1
DynamicHead	88.50%	84.30%	88.40%	77.20%	86.00%
ShareSepHead	88.30%	81.10%	87.50%	75.10%	84.00%
ImplicitHead	90.30%	79.50%	87.30%	75.00%	85.00%
LiteShiftHead	90.40%	80.20%	87.70%	75.30%	85.00%
TransHead	89.90%	80.10%	87.70%	75.20%	85.00%

#### 3.3.3 Selection of classification loss functions

In the task of object detection, the classification loss function is one of the key components in the model training process, directly impacting the model’s ability to classify objects and distinguish between positive and negative samples. Different classification loss functions possess distinct characteristics and applicable scenarios. Focal Loss effectively addresses the issue of class imbalance, while VariFocal Loss further optimizes the focus on positive samples. QFocal Loss and PolyLoss, respectively, enhance the model’s classification performance by introducing new loss calculation methods or combining polynomial functions. To further enhance the detection performance of the sugarcane disease recognition model under the ATSS and DynamicHead detection heads, we conducted a comparative analysis of these four classification loss functions to determine the most suitable loss function for the current task.

The experimental results, as presented in **Table 6**, demonstrate notable performance disparities among four classification loss functions in the task of sugarcane disease recognition. Notably, all four loss functions exhibited consistent performance in terms of F1 score. QFocalLoss emerged as the top performer across precision, recall, mAP50, and mAP50–95 metrics, achieving values of 86.90%, 85.40%, 90.00%, and 77.40%, respectively. This indicates that, under the current model architecture and task, QFocalLoss effectively balances the classification of positive and negative samples, thereby enhancing the model’s detection precision and recall capabilities for disease targets. VariFocalLoss demonstrated comparable performance to QFocalLoss in terms of recall, mAP50, and mAP50-95. Although PolyLoss and FocalLoss also provided satisfactory classification performance, they slightly lagged behind the former two in overall metrics. In summary, it can be concluded that QFocalLoss, when combined with ATSS and DynamicHead, offers the optimal choice of classification loss function for sugarcane disease recognition tasks, contributing to further enhancement of the model’s detection performance and practical application effectiveness.

**Table 6:** Comparison of the performance of four classification loss functions.

Model	Precision	Recall	mAP50	mAP50-95	F1
QFocalLoss	86.90%	85.40%	90.00%	77.40%	86.00%
VariFocalLoss	87.40%	84.70%	89.20%	77.20%	86.00%
PolyLoss	86.50%	84.60%	89.50%	76.90%	86.00%
FocalLoss	87.60%	84.50%	89.70%	76.70%	86.00%

### 3.4 Model comparison

Following the optimization of key components such as the detection head and classification loss function, the YOLOv8m-ATSS-DynamicHead-QFocalLoss (ADQ-YOLOv8m) model structure was derived. To validate the superior detection performance of this enhanced model on the sugarcane disease dataset, a comprehensive comparative analysis was conducted with nine prevalent object detection algorithms. These ten algorithms encompass object detection models with diverse architectures and characteristics, including various versions of the YOLO series (YOLOv8n, YOLOv8s, YOLOv7, YOLOv9, etc.), anchor-based RetinaNet, and region proposal-based Libra R-CNN. During the training process, all models were trained on the same sugarcane disease dataset using identical training parameters to ensure experimental fairness.

The experimental results, as presented in **Table 7**, demonstrate that the ADQ-YOLOv8m model exhibits significant advantages over nine other mainstream object detection algorithms. In terms of detection accuracy, the ADQ-YOLOv8m model outperforms others in precision, recall, mAP50, mAP50-95, and F1 metrics, achieving values of 86.90%, 85.40%, 90.00%, 77.40%, and 86.00%, respectively. This indicates that the ADQ-YOLOv8m model possesses the best comprehensive performance, capable of accurately locating and classifying disease targets while maintaining a consistently high detection accuracy across various detection difficulties. Compared with the latest models yorov12n and yorov13n, adq-yorov8m is superior in all key indicators, especially map50-95, which has increased by 43.7% and 16.0% respectively, showing the excellent detection ability of the model in complex scenes Compared to RetinaNet and Libra R-CNN models, it also demonstrates clear advantages: RetinaNet’s recall value is 1.36% higher than that of the ADQ-YOLOv8m model, yet its mAP50 and mAP50–95 values are both over 20% lower, and its precision is only 19.51%. This suggests that among samples predicted as disease targets, the proportion of true positives is the lowest, and the false alarm rate is the highest. The Libra R-CNN model also performs relatively poorly in various metrics, with precision being over 10% higher than RetinaNet, but sharing similar issues with RetinaNet.

**Table 7:** Comparison of ADQ-YOLOv8m’s performance with other leading object detection algorithms.

Model	Precision	Recall	mAP50	mAP50-95	F1	Depths	Total parameters	FLOPS (G)
ADQ-YOLOv8m	86.90%	85.40%	90.00%	77.40%	86.00%	218	25844392	78.7
YOLOv5n	59.60%	51.50%	54.80%	26.50%	55.00%	262	2510024	7.2
YOLOv5s	66.60%	63.80%	67.60%	36.70%	65.00%	262	9125288	24.1
YOLOv7	63.10%	69.30%	65.10%	29.60%	64.00%	407	37232405	105.2
YOLOv9	76.70%	70.50%	77.20%	48.30%	73.00%	962	51015760	238.9
YOLO11n	65.70%	58.00%	60.90%	33.40%	61.00%	238	2583712	6.3
YOLO11s	78.30%	71.30%	77.70%	51.20%	75.00%	238	9415896	21.3
YOLO11m	82.90%	76.80%	83.50%	61.40%	80.00%	303	20036200	67.7
YOLOv12n	80.18%	76.18%	81.82%	53.85%	78.00%	376	2509904	5.8
YOLOv13n	84.95%	82.04%	87.24%	66.71%	84.00%	535	2449455	6.2
RetinaNet	19.51%	86.76%	77.70%	57.40%	31.85%	50	32314	257.71
Libra R-CNN	30.20%	85.19%	75.90%	49.70%	44.59%	83	41335	133.96

In terms of model complexity and efficiency, ADQ-YOLOv8m boasts a depth of 218, with a parameter count of 25,844,392 and a FLOPS of 78.7G. Despite its relatively large parameter count and computational demands, ADQ-YOLOv8m maintains high performance while exhibiting a more reasonable model complexity compared to sophisticated models such as YOLOv9 (parameter count 51,015,760, FLOPS 238.9G), making it more suitable for deployment and use in practical applications. Compared with the lightweight models yorov12n and yorov13n, the parameters of adq-yorov8m are slightly higher, but its performance is significantly improved. In terms of map50-95, it is about 43.7% and 16.0% higher than yorov12n and yorov13n, respectively, indicating that it is worth increasing the complexity of the model in order to improve the performance in the task of sugarcane disease identification. In addition, compared with some lightweight models such as yorov5n (parameter 2510024, flops 7.2g), the map50–95 of adq-yov8m is significantly ahead, which further verifies its performance. A reasonable balance between advantages and complexity.

**Figure 7:**
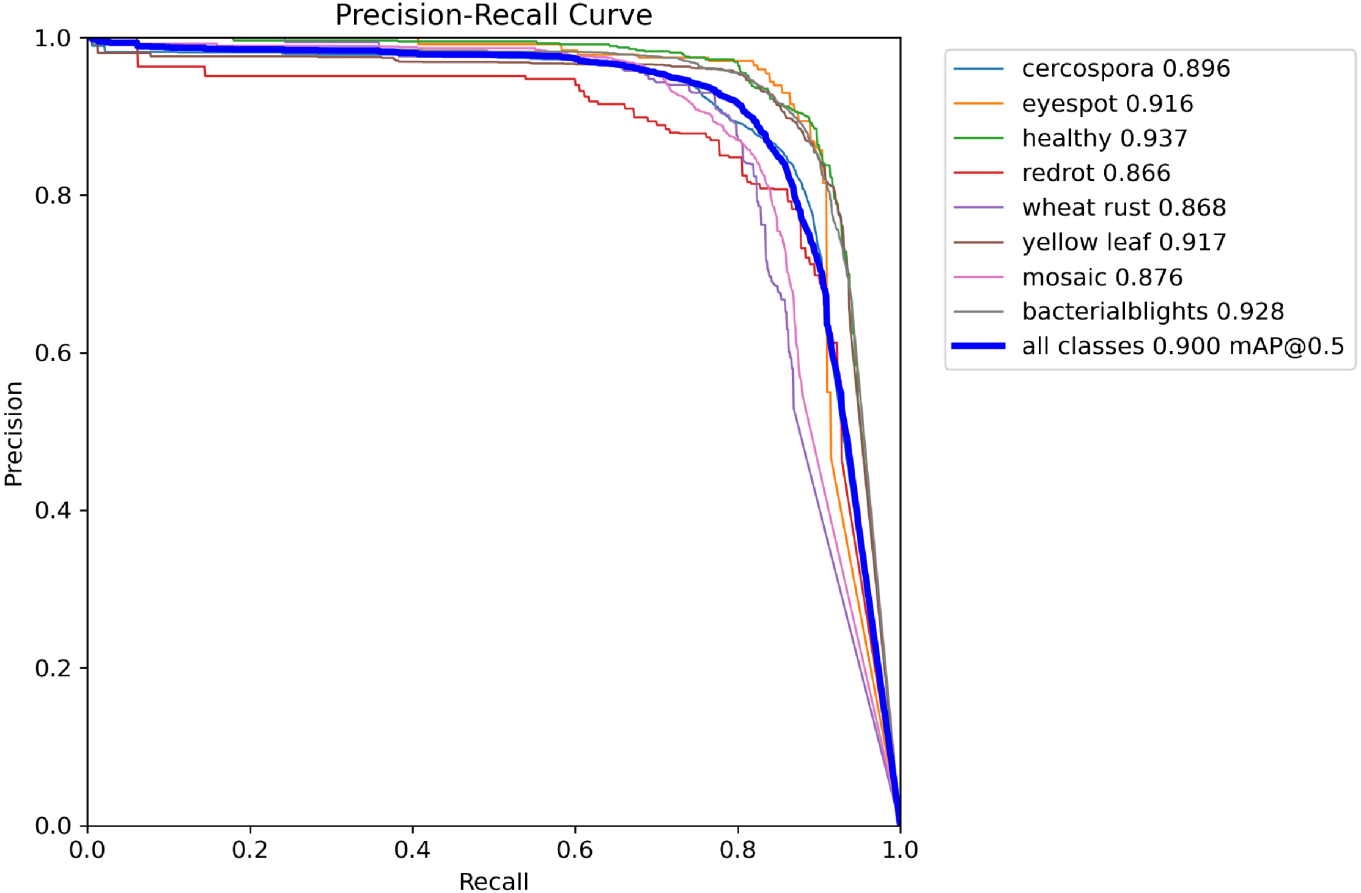
Precision-Recall curves of the ADQ-YOLOv8m model for sugarcane disease detection.

Addressing the issue of sugarcane disease detection, a systematic evaluation and comparison of the performance of 12 different models were conducted in **Table 8**. In terms of detection accuracy, the ADQ-YOLOv8m model demonstrated exceptional performance across most disease categories. Taking Cercospora as an example, its precision reached 86.70%, recall was 84.00%, and mAP50 and mAP50–95 were as high as 89.60% and 76.70%. Compared with YOLOv12n (precision 81.60%, recall 79.30%, map50 84.70%, map50-95 58.60%) and YOLOv13n(precision 86.80%、recall 82.00%、mAP50 89.50%、mAP50-95 68.40%), ADQ-YOLOv8m It increased by 30.89% and 12.13% on map50-95, respectively. In the eyespot category, the precision and recall of adq-YOLOv8m are as high as 90.60% and 86.90%, respectively, while the corresponding indicators of YOLOv5s are 63.30% and 58.30%. Even in the face of diseases such as redrot, which are difficult to detect, the map50 and map50–95 of adq-YOLOv8m still reach 86.60% and 56.90%, which are better than YOLOv12n and YOLOv13n. According to the PR diagram of the model (**Figure 7**), the model shows high precision and recall in all categories, and achieves a good balance. The difference between the highest and lowest accuracy categories is only 7.1%, which further proves the stability and reliability of the model in multi category detection tasks.

**Table 8:** Performance analysis of eleven models across eight detection categories in the fields of artificial intelligence and smart agriculture.

Model	Disease	Precision	Recall	mAP50	mA50-95
ADQ-YOLOv8m	cercospora	86.70%	84.00%	89.60%	76.70%
	eyespot	90.60%	86.90%	91.60%	83.50%
	healthy	89.40%	88.90%	93.70%	80.70%
	redrot	80.30%	86.10%	86.60%	56.90%
	wheat rust	84.10%	81.10%	86.80%	78.70%
	yellow leaf	88.10%	87.90%	91.70%	84.30%
	mosaic	86.70%	80.50%	87.60%	76.60%
	bacterialblights	89.40%	87.50%	92.80%	81.70%
YOLOv5n	cercospora	62.30%	48.70%	56.30%	28.70%
	eyespot	56.80%	46.90%	48.90%	23.10%
	healthy	63.20%	42.00%	53.10%	29.00%
	redrot	60.30%	68.90%	59.80%	22.90%
	wheat rust	60.80%	55.30%	58.40%	28.40%
	yellow leaf	55.80%	56.70%	56.40%	29.90%
	mosaic	56.20%	41.20%	46.30%	21.00%
	bacterialblights	61.70%	52.70%	59.00%	29.00%
YOLOv5s	cercospora	73.30%	70.30%	75.60%	44.70%
	eyespot	63.30%	58.30%	60.10%	33.50%
	healthy	68.70%	52.90%	64.70%	37.20%
	redrot	68.40%	68.30%	67.70%	29.00%
	wheat rust	64.80%	71.20%	74.00%	40.30%
	yellow leaf	65.10%	71.00%	71.20%	42.00%
	mosaic	60.90%	55.40%	59.20%	30.80%
	bacterialblights	67.90%	63.40%	68.40%	36.20%
YOLOv7	cercospora	69.30%	67.80%	71.60%	34.90%
	eyespot	54.20%	58.30%	56.20%	25.10%
	healthy	62.60%	58.70%	63.50%	29.90%
	redrot	67.20%	72.80%	65.90%	25.40%
	wheat rust	64.80%	71.40%	68.70%	30.40%
	yellow leaf	64.60%	69.60%	69.50%	36.10%
	mosaic	57.40%	53.90%	55.70%	24.40%
	bacterialblights	64.70%	67.30%	69.70%	30.90%
YOLOv9	cercospora	80.60%	79.00%	84.60%	58.90%
	eyespot	67.00%	63.80%	69.20%	44.10%
	healthy	79.20%	63.60%	75.50%	48.00%
	redrot	77.40%	72.80%	77.40%	35.30%
	wheat rust	78.30%	77.40%	82.40%	54.70%
	yellow leaf	79.20%	74.20%	80.30%	54.30%
	mosaic	76.70%	59.80%	68.50%	41.00%
	bacterialblights	75.00%	73.40%	79.80%	49.70%
YOLO11n	cercospora	65.30%	55.00%	60.00%	32.80%
	eyespot	60.00%	47.20%	47.30%	25.00%
	healthy	65.30%	51.40%	60.20%	34.00%
	redrot	61.60%	68.30%	61.20%	26.60%
	wheat rust	65.30%	59.80%	62.80%	31.60%
	yellow leaf	71.00%	72.00%	74.70%	52.90%
	mosaic	67.60%	45.50%	52.00%	26.40%
	bacterialblights	69.90%	64.70%	69.20%	37.80%
YOLO11s	cercospora	76.70%	73.70%	77.60%	53.60%
	eyespot	80.60%	67.80%	77.90%	51.20%
	healthy	80.60%	69.20%	79.10%	53.50%
	redrot	75.80%	68.00%	73.00%	33.20%
	wheat rust	79.50%	75.30%	79.00%	52.40%
	yellow leaf	78.80%	77.90%	83.10%	66.80%
	mosaic	77.10%	62.60%	71.10%	47.50%
	bacterialblights	77.00%	75.90%	80.70%	51.20%
YOLO11m	cercospora	81.60%	77.60%	82.80%	63.20%
	eyespot	86.30%	79.10%	87.00%	67.90%
	healthy	84.50%	75.60%	84.80%	63.80%
	redrot	78.20%	75.60%	78.40%	42.20%
	wheat rust	80.20%	79.20%	84.10%	62.90%
	yellow leaf	83.70%	79.20%	85.30%	69.00%
	mosaic	85.50%	68.90%	79.70%	59.70%
	bacterialblights	83.40%	79.30%	86.20%	62.20%
YOLOv12n	cercospora	81.60%	79.30%	84.70%	58.60%
	eyespot	81.20%	75.70%	82.10%	51.80%
	healthy	83.70%	71.20%	82.10%	52.50%
	redrot	83.90%	79.70%	84.20%	39.60%
	wheat rust	77.70%	75.80%	78.50%	50.40%
	yellow leaf	80.30%	80.10%	84.50%	58.40%
	mosaic	78.10%	70.10%	76.80%	48.60%
	bacterialblights	75.10%	77.50%	81.70%	49.40%
YOLOv13n	cercospora	86.80%	82.00%	89.50%	68.40%
	eyespot	82.80%	86.00%	86.80%	60.70%
	healthy	89.20%	82.60%	90.00%	67.00%
	redrot	83.40%	82.80%	88.50%	47.70%
	wheat rust	83.50%	77.40%	83.50%	63.20%
	yellow leaf	86.60%	85.50%	89.10%	69.50%
	mosaic	83.70%	77.40%	82.50%	59.50%
	bacterialblights	83.10%	82.60%	88.20%	61.00%
RetinaNet	cercospora	24.42%	86.40%	55.41%	38.79%
	eyespot	5.97%	89.45%	47.71%	33.39%
	healthy	10.15%	89.17%	49.66%	34.76%
	redrot	8.71%	85.00%	46.85%	32.80%
	wheat rust	14.94%	88.60%	51.77%	36.23%
	yellow leaf	22.48%	90.20%	56.34%	39.44%
	mosaic	14.14%	77.65%	45.90%	32.13%
	bacterialblights	36.52%	90.19%	63.36%	44.35%
Libra R-CNN	cercospora	31.55%	85.16%	73.49%	46.98%
	eyespot	18.43%	87.44%	79.27%	55.22%
	healthy	25.52%	87.04%	75.55%	45.89%
	redrot	34.19%	88.33%	81.41%	48.37%
	wheat rust	32.19%	87.10%	80.34%	57.61%
	yellow leaf	26.17%	89.12%	76.17%	50.01%
	mosaic	19.49%	76.86%	61.04%	38.71%
	bacterialblights	53.33%	86.81%	79.91%	54.85%

In terms of comprehensive performance evaluation, the ADQ-YOLOv8m model exhibits an mAP50 exceeding 86% across various categories, with the mAP50–95 metric surpassing 76% for all categories except redrot. Taking yellow leaf as an example, the ADQ-YOLOv8m achieves an mAP50 of 91.70% and an mAP50–95 of 84.30%, whereas YOLOv7 only attains an mAP50 and mAP50–95 of 69.50% and 36.10% for this category. For the diseases such as bacterialblights that have an important impact on the actual agricultural production, the map50 and map50–95 of adq-yorov8m reached 92.80% and 81.70% respectively, while the corresponding indicators of yorov9 were 75.90% and 49.70%, which were 13.59% and 33.93% higher than yorov12n, and 5.22% and 48.95% higher than yorov13n, respectively.

Through detailed comparative analysis with other models, it is evident that ADQ-YOLOv8m exhibits significant advantages in crop disease detection tasks. Not only does it perform exceptionally well in key metrics such as precision and recall, but it also demonstrates high performance stability across different disease categories. This superior performance is primarily attributed to the improvement of the model’s DynamicHead. Additionally, the model incorporates an ATSS label dynamic allocation strategy and a QFocalLoss loss function in its structure, better accommodating issues such as diverse target sizes and complex morphologies in crop disease detection.

To sum up, the adq-YOLOv8m model performs well in terms of accuracy, recall, detection accuracy and comprehensive performance indicators when compared with other 12 mainstream target detection algorithms. In particular, it shows significant advantages in comparison with YOLOv12n and YOLOv13n, and is the best choice for sugarcane disease identification.

## 4 Discussion

### 4.1 ADQ-YOLOv8m prediction visualization analysis

**Figure 8:**
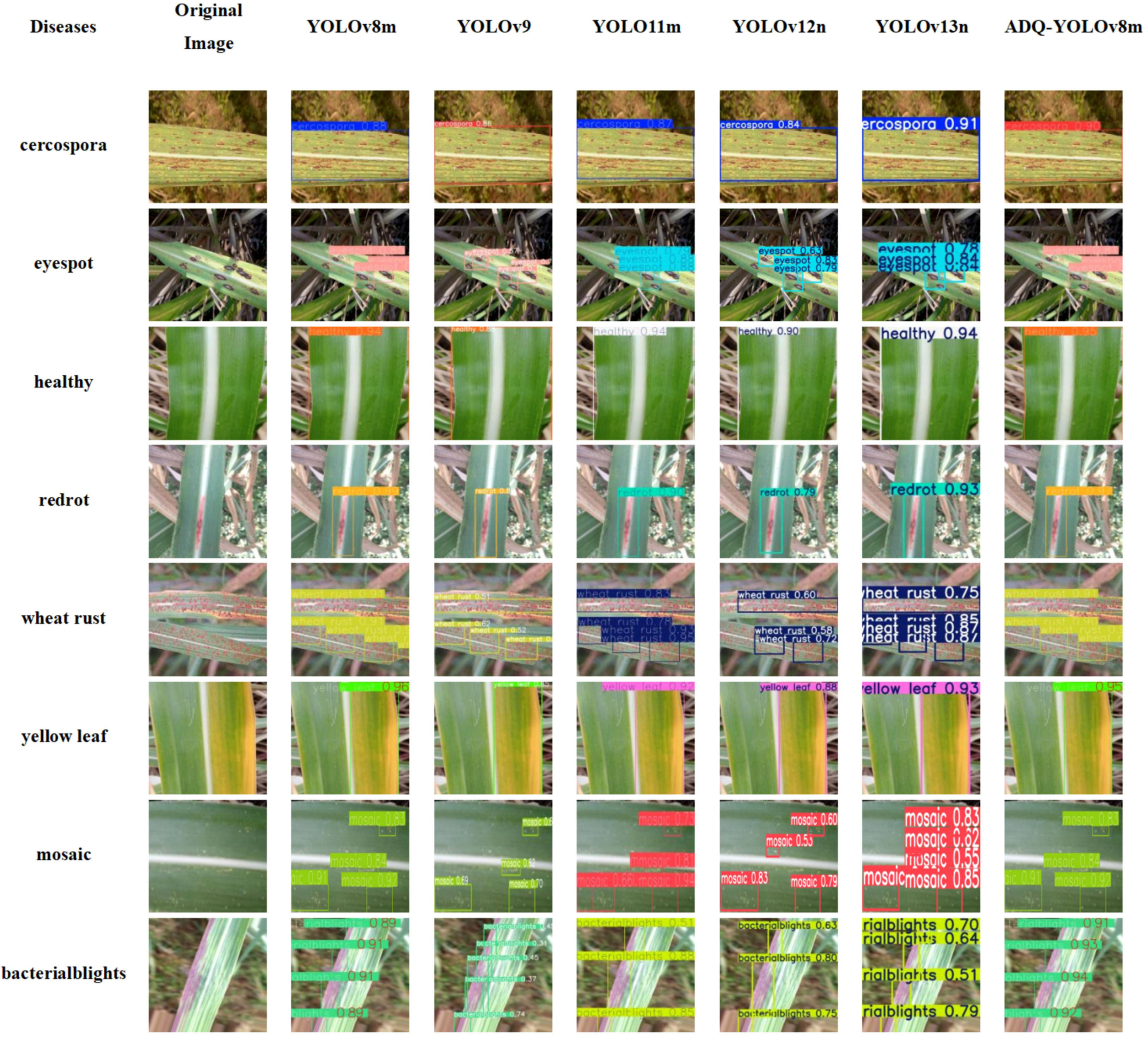
Comparison of detection results among YOLO series models for sugarcane disease detection.

We conducted a comparative analysis of the performance in sugarcane disease detection between the ADQ-YOLOv8m model and other YOLO series models that have demonstrated promising training outcomes. We selected images from eight categories and employed YOLOv8m, YOLOV9, YOLO11N, YOLO11S, YOLO11M, and ADQ-YOLOv8m for predictive analysis. The results are presented in **Figure 8**.

As shown in **Table 9**, ADQ-YOLOv8m shows significant comprehensive advantages, with an average accuracy of 0.915, higher than 0.853 of YOLO11m, and significantly higher than 0.674 of YOLOv9, 0.773 of YOLOv12n, and 0.831 of YOLOv13n. ADQ-YOLOv8m performed stably in most tasks, and the detection accuracy of “healthy” and “red rot” remained stable at 0.95 and 0.97, with stable effects; However, in the multi-target complex scenes, such as “eyespot” and “mosaic”, their accuracy rates are 0.84 and 0.83 – 0.92, respectively, better than YOLOv9’s 0.57-0.7 and 0.62 – 0.7, indicating that they have better effects in multi-target scenes. In contrast, YOLOv9’s accuracy rate in the “when trust” task ranges from 0.51 to 0.62, and the model lacks generalization ability for dense small targets. The accuracy rate of YOLOv12n in the “when trust” task is 0.58 – 0.72 (mean 0.65), slightly better than YOLOv9, but still lower than 0.90 of ADQ-YOLOv8m. The accuracy of YOLOv13n in this task is 0.75 – 0.87 (mean 0.81), but it still does not reach the level of ADQ-YOLOv8m. YOLOv11m’s accuracy rate of 0.92 in the “yellow leaf” task is slightly lower than that of 0.95 in ADQ-YOLOv8m, while YOLOv12n (0.88) and YOLOv13n (0.93) perform stably in this task, but not exceed that of ADQ-YOLOv8m. In a single target detection task (such as “health”), the differences between models are small. ADQ-YOLOv8m, YOLO11m, YOLOv12n and YOLOv13n all reach or close to 0.94, indicating that lightweight models are competitive in low complexity scenarios. The accuracy of YOLOv13n in the “mosaic” task is 0.55 – 0.85 (mean 0.70), lower than 0.83 – 0.92 (mean 0.875) of ADQ-YOLOv8m. ADQ-YOLOv8m is significantly superior to other models in “eyespot” and “bactrialblights” tasks, which further verifies its performance advantages in multi-objective processing and the reasonable balance between performance and complexity.

**Table 9:** Table of detection accuracy of each model.

Model	Cercospora	Eyespot	Healthy	Redrot	Wheat rust	Yellow leaf	Mosaic	Bacterialblights
YOLOv8m	0.88	0.85	0.94	0.99	0.9	0.96	0.83-0.92	0.89-0.91
YOLOv9	0.86	0.57-0.7	0.88	0.8	0.51-0.62	0.85	0.62-0.7	0.31-0.74
YOLO11n	0.87	0.79-0.88	0.94	0.90	0.78-0.95	0.92	0.66-0.94	0.51-0.88
YOLO11s	0.84	0.63-0.83	0.90	0.79	0.58-0.72	0.88	0.53-0.83	0.63-0.80
YOLO11m	0.91	0.78-0.84	0.94	0.93	0.75-0.87	0.93	0.55-0.85	0.51-0.79
ADQ-YOLOv8m	0.90	0.84	0.95	0.97	0.90	0.95	0.83-0.92	0.91-0.94

### 4.2 Cross-scenario adaptability testing of ADQ-YOLOv8m

This section aims to explore the generalization capabilities of the ADQ-YOLOv8m model proposed in this study, ensuring its effectiveness and stability across various crop disease scenarios. On the Roboflow website, we selected two datasets similar to the crop disease recognition task of this study: the Corn Leaf Disease Dataset and the Tomato Disease Dataset. The Corn Leaf Disease Dataset comprises two disease categories, totaling 10,000 images, while the Tomato Disease Dataset includes 15 disease categories, with a total of 3,867 images. Neither dataset underwent image augmentation to ensure that the experimental results accurately reflect the model’s generalization capabilities. The proposed ADQ-YOLOv8m model was employed for generalization experiments, comparing the original and post-training results of the two datasets to analyze their generalization abilities. The training parameters for the generalization experiments were set as follows: image size of 640x640, batch_size of 64, epochs of 500, optimizer of SGD, label smoothness of 0.5, initial learning rate of 0.01, final learning rate of 0.0001, optimizer momentum of 0.937, and optimizer weight decay of 0.0005. The training results are presented in **Table 10**.

**Table 10:** Table of training results for corn and tomato disease datasets.

Dataset	Result	Precision	Recall	mAP50
Corn	Original results	95.00%	96.20%	98.20%
	Training results	99.60%	99.80%	99.40%
Tomato	Original results	46.70%	43.90%	43.60%
	Training results	52.40%	47.80%	47.50%

**Table 10** presents the original and post-training results for two datasets. Specifically, following further training on the corn leaf disease dataset, the model’s precision rose to 99.60%, recall reached 99.80%, and mAP50 stood at 99.40%. These metrics represent improvements of 4.6%, 3.6%, and 1.2% respectively from the original results, indicating the model’s remarkable adaptability and learning capacity in detecting corn leaf diseases. It can further optimize parameters through training to enhance detection accuracy. The tomato disease dataset encompasses a broader range of disease categories, with more intricate feature differences among them, thereby increasing the difficulty of detection. After training, the precision increased to 52.40%, recall reached 47.80%, and mAP50 stood at 47.50%. These metrics represent improvements of 5.7%, 3.9%, and 3.9% respectively from the original results, with a more significant increase compared to the corn leaf disease dataset.

Based on the experimental results above, the ADQ-YOLOv8m model proposed in this study has demonstrated strong generalization capabilities in cross-scenario adaptability tests, making it suitable for applications involving multiple objectives, various categories, and imbalanced categories.

Future research directions are planned to proceed from the following aspects: Firstly, for complex multi-category datasets, we aim to further optimize the model structure to enhance its feature extraction and classification capabilities. Secondly, we intend to explore more effective data augmentation strategies and training methods to improve the model’s adaptability and robustness across different datasets. Thirdly, by integrating prior knowledge and domain-specific information, we will design more targeted model training schemes to address the specific requirements of various crop disease detection tasks.

## 5 Conclusions

In this study, an intelligent model, ADQ-YOLOv8m, for precise detection of sugarcane diseases in complex environments is proposed. A raw dataset containing 6,871 images of sugarcane diseases was constructed based on publicly available datasets. Multiple data augmentation techniques were applied to the data to enhance its diver-sity. By comparing two label assignment strategies, five detection heads, and four classification loss functions, it was concluded that the combination of ATSS with QFocal-Loss and DynamicHead achieved the best performance in sugarcane disease detection. Experimental results indicated that ADQ-YOLOv8m achieved precision, recall, mAP50, mAP50-95, and F1 scores of 86.90%, 85.40%, 90.00%, 77.40%, and 86.00%, respectively, significantly outperforming RetinaNet, Libra R-CNN, and other models in the YOLO series.

Further visual analysis of image prediction and cross-scenario adaptability testing have validated the superior performance of ADQ-YOLOv8m. In image detection, the model achieved an average accuracy of 91.5%, demonstrating significant comprehensive advantages. Meanwhile, through training on datasets of corn leaf diseases and tomato diseases, the model’s precision, recall, and mAP50 metrics improved by 4.6%, 3.6%, and 1.2% (corn dataset) and 5.7%, 3.9%, and 3.9% (tomato dataset) respectively compared to the original results, proving the model’s robust generalization capabilities across different crop disease detection tasks.

This study contributes not only by introducing an efficient, precise, and broadly applicable model for detecting sugarcane diseases, but also by offering a significant technical reference for the identification of diseases in crops belonging to the Poaceae family and sharing similar disease characteristics. The successful application of the ADQ-YOLOv8m model provides robust technical support for intelligent disease monitoring and precise prevention and control in agriculture, promising to significantly mitigate the impact of diseases on crop yield and quality, thereby ensuring the sustainable development of agricultural production.

## Data Availability

The raw data supporting the conclusions of this article will be made available by the authors, without undue reservation.

## References

[B1] Abouelmagd L. M. Shams M. Y. Marie H. S. Hassanien A. E. (2024). An optimized capsule neural networks for tomato leaf disease classification. EURASIP J. Image Video Process. 2024, 2. doi: 10.1186/s13640-023-00618-9

[B2] Abulizi A. Ye J. Abudukelimu H. Guo W. (2024). DM-YOLO: improved YOLOv9 model for tomato leaf disease detection. Front. Plant Sci. 15, 1473928. doi: 10.3389/fpls.2024.1473928, PMID: 40007767 PMC11850125

[B3] Ashwini C. Sellam V. (2024). An optimal model for identification and classification of corn leaf disease using hybrid 3D-CNN and LSTM. Biomed. Signal Process. Control 92, 106089. doi: 10.1016/j.bspc.2024.106089

[B4] Bhargava A. Shukla A. Goswami O. P. Alsharif M. H. Uthansakul P. Uthansakul M.. (2024). Plant leaf disease detection, classification, and diagnosis using computer vision and artificial intelligence: A review. IEEE Access 123, 7443–37469.

[B5] Bala M. Bansal S. (2024). Review—Unveiling the power of deep learning in plant pathology: A review on leaf disease detection. ECS J. Solid State Sci. Technol. 13, 047003. doi: 10.1149/2162-8777/ad3981

[B6] Bao D. Zhou J. Bhuiyan S. A. Adhikari P. Tuxworth G. Ford R.. (2024). Early detection of sugarcane smut and mosaic diseases via hyperspectral imaging and spectral-spatial attention deep neural networks. J. Agric. Food Res. 18, 101369. doi: 10.1016/j.jafr.2024.101369

[B7] Bouacida I. Farou B. Djakhdjakha L. Seridi H. Kurulay M. (2025). Innovative deep learning approach for cross-crop plant disease detection: A generalized method for identifying unhealthy leaves. Inf. Process. Agric. 12, 54–67. doi: 10.1016/j.inpa.2024.03.002

[B8] Chakravarty A. Jain A. Saxena A. K. (2024). “Deep learning approach to sugarcane disease identification: from image analysis to mobile application,” in 2024 4th International Conference on Technological Advancements in Computational Sciences (ICTACS). (Heidelberg: Springer) 1696–1702, A., C., A., J. & A., K. S.

[B9] Dai X. Chen Y. Xiao B. Chen D. Liu M. Yuan L.. (2021). “Dynamic head: Unifying object detection heads with attentions,” in Proceedings of the IEEE/CVF conference on computer vision and pattern recognition. (Piscataway: IEEE) 7373–7382.

[B10] Demilie W. B. (2024). Plant disease detection and classification techniques: a comparative study of the performances. J. Big Data 11, 5. doi: 10.1186/s40537-023-00863-9

[B11] Hang J. Zhang D. Chen P. Zhang J. Wang B. (2019). Classification of plant leaf diseases based on improved convolutional neural network. Sensors 19, 4161. doi: 10.3390/s19194161, PMID: 31557958 PMC6806268

[B12] Huang Y.-K. L. W. Z. R. (2018). Color Illustration of Diagnosis and Control for Modern Sugarcane Diseases, Pests, and Weeds (Singapore: Springer Singapore).

[B13] Kunduracıoğlu İ. Paçal İ. (2024). Deep learning-based disease detection in sugarcane leaves: evaluating efficientNet models. J. Operations Intell. 2, 321–235. doi: 10.31181/jopi21202423

[B14] Kuppusamy A. Sundaresan S. K. Cingaram R. (2024a). Enhancing sugarcane leaf disease classification through a novel hybrid shifted-vision transformer approach: technical insights and methodological advancements. Environ. Monit. Assess. 197, 37. doi: 10.1007/s10661-024-13468-3, PMID: 39643787

[B15] Kuppusamy A. Sundaresan S. K. Cingaram R. (2024b). Enhancing sugarcane leaf disease classification through a novel hybrid shifted-vision transformer approach: technical insights and methodological advancements. Environ. Monit. And Assess. 197, 20. doi: 10.1007/s10661-024-13468-3, PMID: 39643787

[B16] LeCun Y. Bengio Y. Hinton G. (2015). Deep learning. Nature 521, 436–444. doi: 10.1038/nature14539, PMID: 26017442

[B17] Li Z. Sun J. Shen Y. Yang Y. Wang X. Wang X.. (2024). Deep migration learning-based recognition of diseases and insect pests in Yunnan tea under complex environments. Plant Methods 20, 18. doi: 10.1186/s13007-024-01219-x, PMID: 38970029 PMC11229499

[B18] Li X. Wang W. Wu L. Chen S. Hu X. Li J.. (2020). Generalized focal loss: learning qualified and distributed bounding boxes for dense object detection. Adv. In Neural Inf. Process. Syst. 3311, 21002–21012.

[B19] Liu H. Wang Y. Cai T. He K. Tian X. Chen Z.. (2024). Integrated management to achieve synergy in sugarcane production and quality in China. Field Crops Res. 317, 109552. doi: 10.1016/j.fcr.2024.109552

[B20] Lv M. Su W. (2023). YOLOV5-CBAM-C3TR: an optimized model based on transformer module and attention mechanism for apple leaf disease detection. Front. Plant Sci. 14, 1323301. doi: 10.3389/fpls.2023.1323301, PMID: 38288410 PMC10822903

[B21] M., A. M., A., L., K., H. A., N., A. A., B., B. S. M. T., S., M., H., A. M. & R., M (2024). “Edge-cloud remote sensing data-based plant disease detection using deep neural networks with transfer learning,” in IEEE Journal of Selected Topics in Applied Earth Observations and Remote Sensing, (Piscataway: IEEE) Vol. 17. 11219–11229.

[B22] Mangrule R. Afreen K. R. (2024). “Automated sugarcane crop disease forecasting with colour and texture features,” in Computer Methods in Biomechanics and Biomedical Engineering: Imaging & Visualization, (London: Taylor & Francis) vol. 11. 2278907.

[B23] Nawaz M. Nazir T. Javed A. Tawfik Amin S. Jeribi F. Tahir A. (2024). CoffeeNet: A deep learning approach for coffee plant leaves diseases recognition. Expert Syst. Appl. 237, 121481. doi: 10.1016/j.eswa.2023.121481

[B24] Nikarika V. K. N. T. M. R. S. (2024). “An advanced YOLOv7 model for vehicle detection to enhance the security of public places,” in 2024 IEEE International Conference on Information Technology, Electronics and Intelligent Communication Systems (ICITEICS). 1–5.

[B25] Ogrekci S. Unal Y. Dudak M. N. (2023). A comparative study of vision transformers and convolutional neural networks: sugarcane leaf diseases identification. Eur. Food Res. And Technol. 249, 1833–1843. doi: 10.1007/s00217-023-04258-1

[B26] Ong P. Jian J. Li X. Zou C. Yin J. Ma G. (2023). New approach for sugarcane disease recognition through visible and near-infrared spectroscopy and a modified wavelength selection method using machine learning models. Spectrochimica Acta Part A-Molecular And Biomolecular Spectrosc. 302, 11. doi: 10.1016/j.saa.2023.123037, PMID: 37356390

[B27] Pang J. Chen K. Shi J. Feng H. Ouyang W. Lin D. (2019). “Libra r-cnn: Towards balanced learning for object detection,” in Proceedings of the IEEE/CVF conference on computer vision and pattern recognition. 821–830.

[B28] Qaadan S. Alshare A. Ahmed A. Altartouri H. (2025). Stacked ensembles powering smart farming for imbalanced sugarcane disease detection. Appl. Sci. 15, 2788. doi: 10.3390/app15052788

[B29] Rajput S. S. Rai D. Verma H. N. Choudhary R. K. Dwivedi S. S. (2025). MLTSDC: multi level transformer based sugarcane disease classifier. Physiol. And Mol. Plant Pathol. 139, 11. doi: 10.1016/j.pmpp.2025.102799

[B30] Ritharson P. I. Raimond K. Mary X. A. Robert J. E. J A. (2024). DeepRice: A deep learning and deep feature based classification of Rice leaf disease subtypes. Artif. Intell. Agric. 11, 34–49. doi: 10.1016/j.aiia.2023.11.001

[B31] Rott P. C. Girard J. Comstock J. C. (2015). Impact of pathogen genetics on breeding for resistance to sugarcane diseases. Int. Sugar J. 117, 494–499.

[B32] Sangaiah A. K. Yu F. N. Lin Y. B. Shen W. C. Sharma A. (2024). UAV T-YOLO-rice: an enhanced tiny yolo networks for rice leaves diseases detection in paddy agronomy. IEEE Trans. Network Sci. Eng. 11, 5201–5216. doi: 10.1109/TNSE.2024.3350640

[B33] Srinivasan S. Prabin S. M. Mathivanan S. K. Rajadurai H. Kulandaivelu S. Shah M. A. (2025). Sugarcane leaf disease classification using deep neural network approach. BMC Plant Biol. 25, 282. doi: 10.1186/s12870-025-06289-0, PMID: 40033192 PMC11877950

[B34] Srivastava S. Kumar P. Mohd N. Singh A. Gill F. S. (2020). A novel deep learning framework approach for sugarcane disease detection. SN Comput. Sci. 1, 87. doi: 10.1007/s42979-020-0094-9

[B35] Sun J. Li Z. Li F. Shen Y. Qian Y. Li T. (2024). EF yolov8s: A human–computer collaborative sugarcane disease detection model in complex environment. Agronomy 14, 2099. doi: 10.3390/agronomy14092099

[B36] Trinh D. C. Mac A. T. Dang K. G. Nguyen H. T. Nguyen H. T. Bui T. D. (2024). Alpha-EIOU-YOLOv8: an improved algorithm for rice leaf disease detection. AgriEngineering 6, 302–317. doi: 10.3390/agriengineering6010018

[B37] Vallabhajosyula S. Sistla V. Kolli V. K. K. (2024). A novel hierarchical framework for plant leaf disease detection using residual vision transformer. Heliyon 10(9), e29912. doi: 10.1016/j.heliyon.2024.e29912, PMID: 38699004 PMC11064133

[B38] Wang X. Liu J. (2024). Vegetable disease detection using an improved YOLOv8 algorithm in the greenhouse plant environment. Sci. Rep. 14, 4261. doi: 10.1038/s41598-024-54540-9, PMID: 38383751 PMC10881480

[B39] Wang J. Qin C. Hou B. Yuan Y. Zhang Y. Feng W. (2024). LCGSC-YOLO: a lightweight apple leaf diseases detection method based on LCNet and GSConv module under YOLO framework. Front. Plant Sci. 15, 1398277. doi: 10.3389/fpls.2024.1398277, PMID: 39544536 PMC11560749

[B40] Wang Y. Wang C. Zhang H. Dong Y. Wei S. (2019). Automatic ship detection based on RetinaNet using multi-resolution Gaofen-3 imagery. Remote Sens. 11, 531. doi: 10.3390/rs11050531

[B41] Wang C. Yeh I. Mark Liao H. (2024). “Yolov9: Learning what you want to learn using programmable gradient information,” in European conference on computer vision (London: Springer), 1–21.

[B42] Wang Y. Yin Y. Li Y. Qu T. Guo Z. Peng M.. (2024). Classification of plant leaf disease recognition based on self-supervised learning. Agronomy 14(3), 500. doi: 10.3390/agronomy14030500

[B43] Yao J. Qi J. Zhang J. Shao H. Yang J. Li X. (2021). A real-time detection algorithm for kiwifruit defects based on YOLOv5. Electronics 10, 1711. doi: 10.3390/electronics10141711

[B44] Yao J. Tran S. N. Garg S. Sawyer S. (2024). Deep learning for plant identification and disease classification from leaf images: multi-prediction approaches. ACM Comput. Surv. 56, 153. doi: 10.1145/3639816

[B45] Yaseen M. (2024). What is YOLOv8: An in-depth exploration of the internal features of the next-generation object detector. arXiv.

[B46] Zhang T. Luo B. Sharda A. Wang G. (2022). Dynamic label assignment for object detection by combining predicted ioUs and anchor ious. J. Imaging. 8(7), 193. doi: 10.3390/jimaging8070193, PMID: 35877638 PMC9322857

