# Correction: ADQ-YOLOv8m: a precise detection model of sugarcane disease in complex environment

**DOI:** 10.3389/fpls.2025.1725871

**Published:** 2025-10-27

**Authors:** Zhaowen Li, Jihong Sun, Ying Yang, Jiangquan Chen, Qian Yu, Zheng Zhou, Yan Yang, Tao Yin, Haokai Zhang, Ye Qian

**Affiliations:** ^1^ College of Big Data, Yunnan Agricultural University, Kunming, China; ^2^ Key Laboratory of Artificial Intelligence in Yunnan Province, Kunming University of Science and Technology, Kunming, Yunnan, China; ^3^ School of Information Engineering, Kunming University, Kunming, China; ^4^ College of Animal Veterinary Medicine, Yunnan Agricultural University, Kunming, China; ^5^ National Pilot School of Software, Yunnan University, Kunming, China; ^6^ Scientific and Technological Achievements Transfer and Transformation Center, Yunnan Provincial Academy of Science and Technology, Kunming, China; ^7^ Engineering College, China Agricultural University, Beijing, China

**Keywords:** complex environment, sugarcane diseases, YOLOv8, precise detection, generalization ability

Authors “Jiangquan Chen, Tao Yin, and Ye Qian” were erroneously assigned to affiliation 2 “Key Laboratory of Artifcial Intelligence in Yunnan Province, Kunming University of Science and Technology, Kunming, Yunnan, China”. This affiliation has now been removed for authors “Jiangquan Chen, Tao Yin, and Ye Qian”.

The original version of this article has been updated.

